# An Evaluation of the Effect of App-Based Exercise Prescription Using Reinforcement Learning on Satisfaction and Exercise Intensity: Randomized Crossover Trial

**DOI:** 10.2196/49443

**Published:** 2024-11-26

**Authors:** Cailbhe Doherty, Rory Lambe, Ben O’Grady, Diarmuid O’Reilly-Morgan, Barry Smyth, Aonghus Lawlor, Neil Hurley, Elias Tragos

**Affiliations:** 1 School of Public Health, Physiotherapy and Sports Science, University College Dublin, Dublin, Ireland; 2 Insight SFI Research Centre for Data Analytics, O'Brien Centre for Science, University College Dublin, Dublin, Ireland

**Keywords:** reinforcement learning, exercise therapy, personal satisfaction, satisfaction, physiotherapy, physical therapy, exercise intensity, mobile apps, randomized controlled trial, crossover trial, apps, exercise, physical activity, mobile phone

## Abstract

**Background:**

The increasing prevalence of sedentary lifestyles has prompted the development of innovative public health interventions, such as smartphone apps that deliver personalized exercise programs. The widespread availability of mobile technologies (eg, smartphone apps and wearable activity trackers) provides a cost-effective, scalable way to remotely deliver personalized exercise programs to users. Using machine learning (ML), specifically reinforcement learning (RL), may enhance user engagement and effectiveness of these programs by tailoring them to individual preferences and needs.

**Objective:**

The primary aim was to investigate the impact of the Samsung-developed i80 BPM app, implementing ML for exercise prescription, on user satisfaction and exercise intensity among the general population. The secondary objective was to assess the effectiveness of ML-generated exercise programs for remote prescription of exercise to members of the public.

**Methods:**

Participants were randomized to complete 3 exercise sessions per week for 12 weeks using the i80 BPM mobile app, crossing over weekly between intervention and control conditions. The intervention condition involved individualizing exercise sessions using RL, based on user preferences such as exercise difficulty, selection, and intensity, whereas under the control condition, exercise sessions were not individualized. Exercise intensity (measured by the 10-item Borg scale) and user satisfaction (measured by the 8-item version of the Physical Activity Enjoyment Scale) were recorded after the session.

**Results:**

In total, 62 participants (27 male and 42 female participants; mean age 43, SD 13 years) completed 559 exercise sessions over 12 weeks (9 sessions per participant). Generalized estimating equations showed that participants were more likely to exercise at a higher intensity (intervention: mean intensity 5.82, 95% CI 5.59‐6.05 and control: mean intensity 5.19, 95% CI 4.97‐5.41) and report higher satisfaction (RL: mean satisfaction 4, 95% CI 3.9-4.1 and baseline: mean satisfaction 3.73, 95% CI 3.6-3.8) in the RL model condition.

**Conclusions:**

The findings suggest that RL can effectively increase both the intensity with which people exercise and their enjoyment of the sessions, highlighting the potential of ML to enhance remote exercise interventions. This study underscores the benefits of personalized exercise prescriptions in increasing adherence and satisfaction, which are crucial for the long-term effectiveness of fitness programs. Further research is warranted to explore the long-term impacts and potential scalability of RL-enhanced exercise apps in diverse populations. This study contributes to the understanding of digital health interventions in exercise science, suggesting that personalized, app-based exercise prescriptions may be more effective than traditional, nonpersonalized methods. The integration of RL into exercise apps could significantly impact public health, particularly in enhancing engagement and reducing the global burden of physical inactivity.

## Introduction

Every year, hundreds of randomized controlled trials evaluating the effects of exercise in diverse population groups are published. This research has demonstrated the medicinal capacities of exercise for numerous chronic diseases [1]. Consequently, exercise is considered an essential component in the management plans of many health care interventions [1-3]. Regular exercise has also been shown to have wide-ranging health benefits for nonpathological groups; for instance, there is evidence to suggest that a sedentary lifestyle may be an even stronger predictor of mortality than smoking, hypertension, and diabetes [4]. National and international campaigns, advertisements, and public health guidelines have been developed to increase public awareness of the benefits of exercise [5,6]. Despite this, inactivity (and the increased risk of morbidity and mortality associated with it) remains a significant public health concern [7,8]. This raises the question: if people know that regular physical activity and exercise are good for them, why are so many inactive?

Even in randomized controlled trials, low levels of adherence to exercise sometimes belie the treatment effect to such an extent that results in comparison to a nonexercising control group are statistically insignificant [9,10]. Adherence to exercise, which is defined as the degree to which the target intensity and volume are achieved [11,12], is likely to be worse among the general population, who are not enthusiastic volunteers in a research study and who are being closely supervised by a research team [13].

Many theories and models have been proposed to explain why adherence to exercise is suboptimal [14,15]. A recent umbrella review identified a number of key factors for improving adherence to exercise [16]. Among the 14 factors that were identified, individualizing the exercise program, making sure that it integrates easily into participants’ daily living schedules, continually monitoring and providing feedback on progress (and adapting the exercise program accordingly), ensuring that users have an active role in goal setting, and using technology to deliver the exercise intervention were deemed to be important in improving adherence [16]. Patient education was also considered crucial to increase self-efficacy, enhancing the knowledge about what they can do and what they can change to improve their overall health [17-19].

Mobile technologies (eg, smartphone apps and wearable activity trackers) are a cost-effective, scalable way of delivering exercise programs to users that incorporate these factors. Over 60% of adults worldwide own a smartphone, with worldwide penetration rates highest in the United States (where >80% of the population uses a smartphone) [20]. In addition to being able to deliver interventions through wireless internet and messaging connectivity, smartphones have in-built tools like global positioning systems, inertial measurement units, and cameras that can objectively measure several exercise parameters [21-23]. Smartphones also have powerful computation and communication capabilities that enable the use of machine learning (ML) and artificial intelligence to individualize each user’s exercise program.

Reinforcement learning (RL) represents a compelling method within ML for tailoring and adapting exercise programs to individual users. In RL, a decision-making agent performs actions that lead to preferred states within its environment. Each action transitions the environment to a new state, following which the agent receives feedback—either positive or negative reinforcement. This feedback helps to refine the agent’s “policy,” which is essentially a strategy that maps states to actions aimed at maximizing cumulative rewards over time.

For instance, consider the use of RL within a smartphone app designed for exercise prescription. Here, the agent could consider variables such as user satisfaction or perceived exertion (ie, the session’s intensity) as states. The agent’s actions could involve adjusting various exercise parameters, such as the number of sets, repetitions, or rest periods in a training session, or altering the types of recommended exercises. The reward function would assess the degree of user satisfaction or exertion, aiming to minimize discrepancies and optimize user experience.

RL is particularly well-suited to the sequential decision-making required in exercise prescription. It operates in a cycle where the agent proposes an exercise session, the user completes it and provides feedback, and the agent uses this information to tailor future sessions. Although several frameworks exist for automating exercise prescription based on user demographics, fitness levels, engagement behaviors, and preferences [24-27], comprehensive studies evaluating the effectiveness of fully computerized, app-based exercise prescription remain limited [28-31]. This gap underscores the need for further research to validate and optimize RL apps in this field.

Therefore, the aim of this study was to test a smartphone app that generates adaptive exercise regimes, incorporating RL to personalize the composition of exercises within sessions based on user satisfaction. We compared user satisfaction between sessions generated by the RL system to a control condition that administered a generic exercise to the user, irrespective of their preferences. The primary outcome for this aim was users’ satisfaction with exercise, which was defined by an abbreviated 8-item version of the Physical Activity Enjoyment Scale (PACES-8) [32,33].

Our primary hypotheses were that users would report higher satisfaction and demonstrate greater adherence to exercise programs generated using RL compared with sessions that are generated randomly or using predefined (ie, nonpersonalized) templates. Our secondary hypothesis was that users would demonstrate higher levels of exertion, measured using Borg’s rate of perceived exertion (RPE) scale, during sessions generated with an RL approach.

## Methods

### Study Design

**Figure 1.**
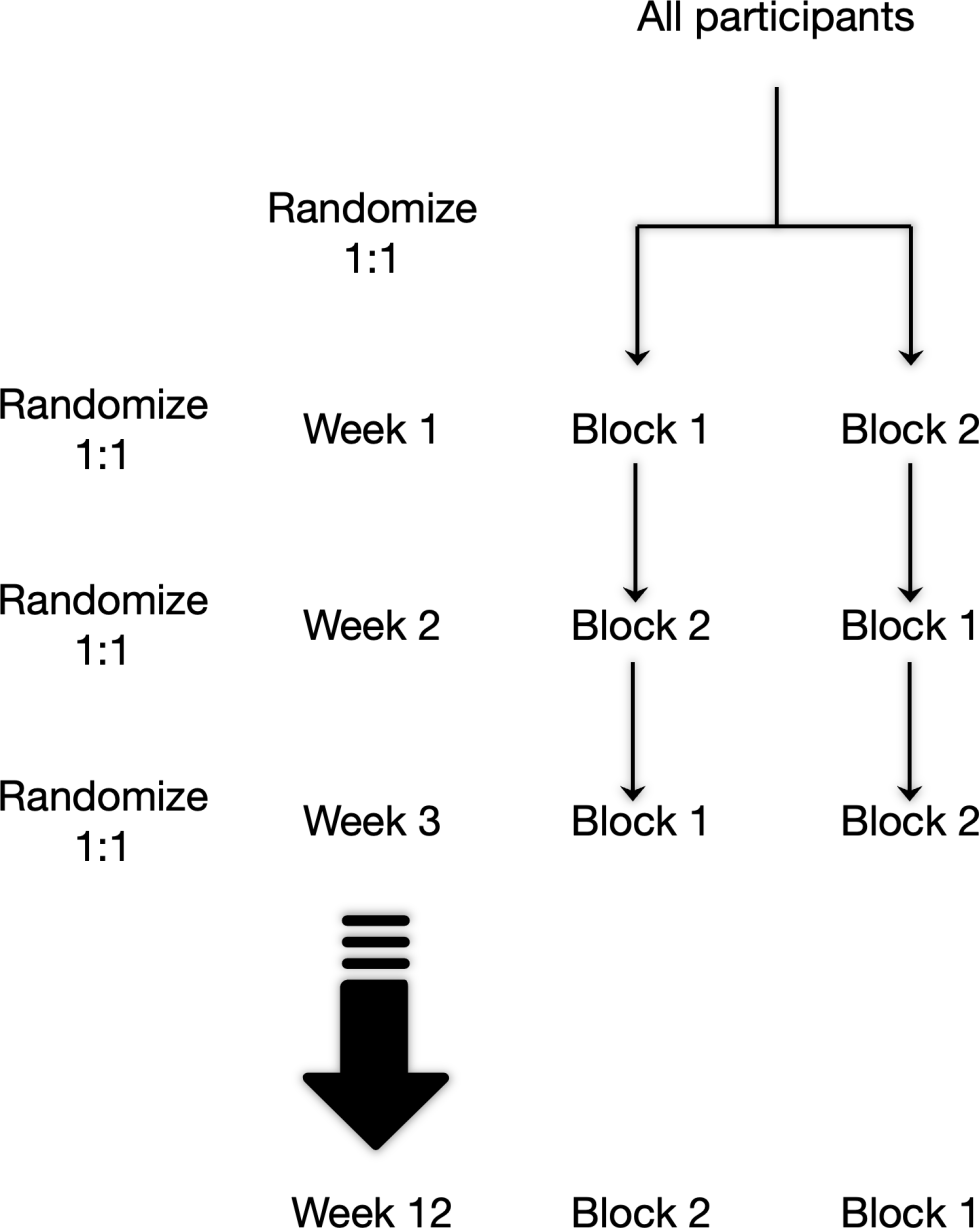
Design of the trial.

The protocol for this study was developed using the SPIRIT (Standard Protocol Items: Recommendations for Interventional Trials) checklist [34] The design was a 12-week, assessor-blinded, randomized crossover trial, with the primary end point being user satisfaction after each exercise session. However, unlike a typical crossover trial, participants transitioned “back and forth” between experimental conditions. Specifically, each participant alternated between the intervention and control conditions at the end of each 1-week cycle. The order in which participants completed each condition (RL condition and control condition) was altered on a weekly basis; each participant was administered condition-generated exercise sessions for 1 week. Each of these 1-week cycles was comprised of 3 workout sessions of approximately 20 minutes in duration, containing >30 exercises (each exercise lasted <30 seconds). The only difference between the workout sessions within each condition was the specific exercises that were recommended (and the order in which they were completed); all other parameters of exercise were held consistent. After a 1-week cycle had elapsed, each cluster of participants completing the protocol under each of the 2 conditions crossed over to the opposing condition, with each crossover marking the start of a new 1-week cycle, regardless of whether participants actually completed the sessions within that cluster. This design is illustrated in Figure 1.

### Ethical Considerations

The University College Dublin Human Research Ethics Committee approved this study (LS-21‐34-Tragos-Lawlor). Written informed consent was obtained, and health screening was conducted for all participants before they enrolled in the trial. The trial was not prospectively registered. Recruitment was conducted between July 6, 2022, and August 29, 2022, and the trial was completed on November 16, 2022. During data collection, each participant was given a participant ID number when they registered with the app. The data were stored using these ID numbers. The key to the code that matched participants’ full name to their ID number was saved in an electronic password-protected file, which was stored on an encrypted drive. The authorship team had sole access rights to these key codes and ID numbers during the trial. Data acquired via the smartphone app were stored on a password-protected University College Dublin server. After data collection, data were anonymized by permanently deleting the file with the participants’ identification key. Participants were not compensated for their participation.

### Population

Participants were recruited from Dublin, Ireland, and its environs via word of mouth and social media. Male and female participants aged 18 to 65 years were recruited. To be eligible for inclusion, participants were required to be healthy, recreationally active adults. This was defined as having engaged in aerobic activity for a total of 80 minutes at moderate intensity, less than or equal to twice per week. Prior to randomization, participants were screened for eligibility by the “exercise preparticipation health screening questionnaire for exercise professionals.” Exclusion criteria included an inability to exercise due to physical disability or motor impairment, having a severe cognitive impairment, or an inability to read and write in English.

### Blinding

Participants were blinded to their allocation throughout the course of the trial. The researcher conducting data analysis was also blinded to knowledge of the intervention.

### Procedure

Upon expressing interest, potential participants received detailed information about the trial along with a health screening questionnaire. After completing the health screening, participants attended an information session via Zoom (Zoom Video Communications), where they were shown how to use the app and its various functions and also instructed on downloading it to their mobile devices. At this session, informed consent was obtained. Each participant was issued a Samsung Galaxy Fit 2 smartwatch (Samsung Electronics Co, Ltd) to use during the exercise sessions for recording heart rate data. Several studies have demonstrated the accuracy of the Samsung Gear Fit for heart rate measurement during a variety of activity types, making it a suitable choice for this study [35-37]. Participants were allowed to keep the fitness tracker after the trial concluded. Immediately following randomization, baseline data collection was carried out through the app.

### Interventions

Participants were asked to complete 3 exercise sessions weekly over 12 weeks using the i80 BPM smartphone app developed by Samsung Electronics Co, Ltd. This app offers video-guided exercise programs and includes a library of 161 exercises. Using RL, i80 BPM customizes and adapts exercise sessions to suit the user’s preferences and abilities. The duration of these sessions, ranging from 10 to 30 minutes, was selected by the user. Users could create personalized session plans varying from 1 to 12 weeks and choose between aerobic and muscle-strengthening exercises, targeting specific muscle groups. The RL model selected exercises for each session based on these preferences, ensuring a diverse range of possible sessions.

Aside from the RL model’s app, all other parameters remained constant across both the intervention and control conditions. These included the exercises available, the app’s interface, and options for session duration and focus (aerobic vs muscle strengthening). The key difference was the individualization of exercise sessions using the RL model in the intervention condition, whereas the control condition used generic, nonpersonalized sessions. Further details on the RL model are discussed elsewhere [38], but in summary, the model used within the i80 BPM app aimed to optimize user satisfaction by personalizing the exercise sequence based on user feedback and evolving preferences. The app’s RL framework used a decision-making agent within an environment shaped by user interactions and fitness profiles. The model was trained to maximize expected rewards, which incorporated metrics of performance and user feedback. In this framework, “actions” referred to the selection of exercises from the app’s database. The “state” included information such as the user’s fitness level and exercise preferences along with session specifics like previously recommended exercises and user feedback. The “reward function” balanced various elements including exercise diversity, suitability to the user’s fitness level, and feedback after each session to ensure that the recommendations met the user’s goals and responses to past workout sessions. The RL model used a neural network to map states to action probabilities, optimizing the sequence of recommended exercises to maximize cumulative rewards—reflecting both user satisfaction and session effectiveness [38].

### RL Intervention

The RL intervention used the app’s full functionality, meaning that exercise sessions were tailored to the user by the RL model, as outlined earlier.

### Baseline Control Condition

The control condition used the same i80 BPM app as described earlier; however, the RL model was not applied. Instead, generic exercise sessions were provided for the user, irrespective of their preferences.

### Crossover Design

As crossover design was used in this trial, participants transitioned between the intervention and control conditions. At the end of each 1-week cycle, participants transitioned to the opposing condition regardless of how many sessions they had completed. Therefore, each participant could complete exercise sessions that were adapted by the RL model (intervention) as well as sessions that were not adapted (control). This design was chosen to maximize the amount of data captured for each condition in anticipation of a high number of dropouts or waning adherence as the trial progressed.

### Outcome Measures

Our primary outcome, user satisfaction, was determined after each session and was collected via the smartphone app. Satisfaction was measured using an abbreviated PACES-8 [32,33].

Our secondary outcome was perceived exertion, measured using the Borg scale, which was administered via the smartphone app at the end of each session; participants were cued to “rate your perceived exertion on a scale from 2 to 10, where 2 means ‘really easy’ and 10 means ‘maximal exertion.’” Additionally, heart rate data were collected using the Galaxy Fit 2 smartwatch provided to each participant [37]. This device uses photoplethysmography to measure heart rate continuously during the exercise sessions. The heart rate data captured by the smartwatch were automatically relayed to the i80 BPM app, which was used to record and analyze these measurements as part of the session data. See Table 1 for all outcomes and questionnaires.

**Table 1.** Outcome measures.

		Baseline	Weekly	During each session	After each session
**Satisfaction**					
	Physical Activity Enjoyment Scale-8				✓
	Rating of perceived exertion				✓
	Heart rate			✓	
	Exercise skipped			✓	
**Behavioral engagement**					
	Exercise altered			✓	
	Total time spent on the app		✓		
	Number of sessions completed		✓		
**User profiling**					
	Demographics (eg, age and sex)	✓			
	International Physical Activity Questionnaire	✓			

### Public Involvement

During an initial feasibility trial, members of the public worked with us to evaluate the sessions generated by the RL system and the mobile app user interface and were asked to assess the burden and time commitment of the study as part of a user-centered design approach [19]. During this feasibility study, participants also completed a web-based survey to establish their current activity levels, their experience with different health and fitness smartphone apps, how they currently exercise, and their self-efficacy. Participants were then asked to use the app over an 8-week period (between June and October 2021) and were asked to provide feedback on their experience. Analysis of participants’ feedback and the app use logs was undertaken by the project team, and the app was further iterated based on this feedback. The version of the app that was used in this study (which took place between June and October 2022) integrated all participants’ feedback from this initial feasibility trial.

### Power Calculations and Sample Size

A sample size of minimum 40 participants was estimated based on the following factors: the sample size recruited as part of the initial feasibility study (we previously recruited 36 participants over an 8-week trial period), an estimated mean difference of 8 on the PACES-8 scale based on previous work for 80% power, and the rule of thumb of at least 10 events for variable (or measures of user satisfaction and behavioral and physiological engagement) [39]. Controlling for 15% dropout, we aimed to recruit a total of 42 participants.

### Data Management and Statistical Analysis

#### Overview

All collected data were anonymized before analysis, with each participant assigned a unique identification code that was used in place of personal identifiers. Data were stored on secure, encrypted servers accessible only to authorized personnel involved in the study. All electronic communications and data transmissions involving participant information were encrypted. Participant consent forms and other sensitive documents were stored separately from the study data, in locked cabinets within secure facilities.

#### Baseline Characteristics

Descriptive statistics were used to summarize user demographics and scores on the questionnaires.

#### Primary and Secondary Hypotheses

The primary outcome measure, user satisfaction, was assessed using the PACES-8 questionnaire, while the secondary outcome, perceived exertion, was measured using the Borg scale. Both outcomes were evaluated at the session level, meaning each exercise session contributed to the analysis rather than summarizing data at the participant level.

To account for the repeated measures design and the correlation of observations within participants, generalized estimating equations (GEEs) were used to assess differences between the RL and control conditions for both primary and secondary outcomes. The dependent variables in the models were PACES satisfaction scores and Borg perceived exertion scores. The independent variables included condition (RL vs control) and trial week (treated as a continuous variable to account for time effects). Covariates adjusted for in the models included age, gender, and baseline physical activity level (categorized as low, moderate, or high). User ID was included as a subject effect to account for within-subject correlation. An independent working correlation matrix was specified for the GEE models, with an identity link function used for both models. Normal distribution was assumed for the outcomes. Model fit was assessed using the quasi-likelihood under independence model criterion and the corrected quasi-likelihood under independence model criterion, with lower values indicating better model fit. The main effects of condition and trial week were estimated, and their significance was assessed using Wald chi-square tests. All statistical analyses were performed using SPSS Statistics software (version 29; IBM Corp).

## Results

In total, 69 participants were initially recruited for the study (27 male and 42 female participants); however, only 62 (24 male and 38 female participants) completed at least 1 exercise session. The mean age of participants was 42.8 (SD 13.3) years, with an average height of 1.7 (SD 2.4) m, body mass of 75.7 (SD 21.1) kg, and BMI of 25.8 (SD 7.8) kg/m^2^. These 62 participants completed 559 exercise sessions between them (9 sessions per participant). A total of 16% (n=11) adhered to at least 50% (n=18) of the sessions they were assigned to complete (ie, 18 of 36), while 34% (n=23) adhered to at least 25% (n=9) of sessions. In total, 268 sessions were completed that were comprised of exercises generated by the RL model, while 291 sessions were completed that were comprised of exercises generated by the baseline model.

The PACES-8 GEE estimated a main effect for condition. An analysis of the parameter estimates revealed that participants were more satisfied with exercise sessions generated by the RL model (RL condition: mean satisfaction 4, 95% CI 3.9-4.1 and baseline condition: mean satisfaction 3.73, 95% CI 3.6-3.8). The main effect for condition was significant (*P*=.02), with a parameter estimate of B=−0.261 (SE 0.109), indicating a modest but meaningful difference in satisfaction scores. The 95% CI for this estimate ranged from −0.475 to −0.047.

The intensity GEE estimated significant main effects for condition (*P*<.01) and trial week (*P*=.048). The intensity was significantly higher in sessions generated by the RL model compared to the baseline model (RL condition: mean intensity 5.82, 95% CI 5.59‐6.05 and baseline condition: mean intensity 5.19, 95% CI 4.97‐5.41). Analysis of the parameter estimates indicated that participants exercised at higher intensities during RL sessions (B=−0.633, SE 0.179; *P*<.01).

We conducted an exploratory analysis of the separate questions from the PACES-8 questionnaire. Specifically, separate GEEs assessed differences in responses to individual items on the PACES-8 questionnaire. Significant main effects for condition were found for items related to pleasure (item #1), invigoration (item #4), gratification (item #5), exhilaration (item #6), stimulation (item #7), and how refreshing each session was (item #8; *P*=.02 for each). Summary statistics for the primary outcome measures of satisfaction, difficulty, heart rate, the number of exercises completed, and the duration for exercise sessions generated by the baseline and RL models are presented in Table 2.

**Table 2.** Summary statistics for overall satisfaction and heart rate for the baseline and reinforcement learning (RL) models and the average scores for the individual PACES-8^a^ items.

Outcome	Baseline, mean (95% CI)	RL model, mean (95% CI)	*P* value
PACES-8	3.73 (3.6-3.8)	4 (3.9-4.1)	.02
Heart rate	106.81 (105-107)	107.24 (106-108)	—^b^
Intensity	5.19 (4.97-5.41)	5.82 (5.59-6.05)	<.01
Duration (minutes)	17 (17-18)	20 (20-21)	—
PACES-8_pleasure^c^	3.66 (3.6-3.7)	3.88 (3.8-4.0)	.047
PACES-8_fun^d^	3.53 (3.4-3.6)	3.72 (3.6-3.8)	.08
PACES-8_pleasant^e^	3.53 (3.4-3.6)	3.71 (3.6-3.8)	.05
PACES-8_invigorating^f^	3.75 (3.7-3.8)	4.15 (4.1-4.2)	<.01
PACES-8_gratifying^g^	3.84 (3.7-3.9)	4.17 (4.1-4.3)	<.01
PACES-8_exhilarating^h^	3.74 (3.6-3.8)	4.04 (3.9-4.1)	.02
PACES-8_stimulating^i^	3.85 (3.7-3.9)	4.13 (4.0-4.2)	.02
PACES-8_refreshing^j^	3.84 (3.7-3.9)	4.06 (4.0-4.1)	.02

a PACES-8: 8-item version of the Physical Activity Enjoyment Scale.

b Not available.

c PACES-8_pleasure: item related to the pleasure derived from the exercise.

d PACES-8_fun: item related to the fun experienced during the exercise session.

e PACES-8_pleasant: item related to how pleasant the exercise session felt.

f PACES-8_invigorating: item related to the invigorating nature of the exercise session.

g PACES-8_gratifying: item related to the gratification from the exercise session.

h PACES-8_exhilarating: item related to the exhilaration felt during the exercise session.

i PACES-8_stimulating: item related to how stimulating the exercise session was.

j PACES-8_refreshing: item related to how refreshing the exercise session felt.

## Discussion

### Principal Findings

The aim of this randomized crossover trial was to investigate whether exercise sessions generated using RL were associated with better user satisfaction compared with a control condition. In this trial, exercise recommendations were delivered via the same smartphone app over the course of a 12-week “back and forth” crossover period, in which participants were subjected to each experimental condition in a 1-week cycle, each comprising 3 exercise sessions of 20 minutes in duration. Whether participants actually completed the sessions within a cycle did not affect their weekly crossover to an alternative condition. The primary outcome of user satisfaction was determined using the PACES-8 questionnaire, which has been previously used in diverse population groups [32,33,40,41] and as part of smartphone app testing protocols [42]. The secondary outcome, perceived exertion, was determined after each exercise session using the Borg scale.

During the trial, 62 (62% female) participants completed 559 exercise sessions (approximately 9 sessions per participant). Our results showed that participants were more satisfied with exercise sessions generated by the RL model (RL condition: mean satisfaction 4, 95% CI 3.9-4.1 and baseline condition: mean satisfaction 3.73, 95% CI 3.6-3.8). By incorporating trial week as a covariate in our analysis, we were able to show that participants were generally more satisfied with exercise sessions generated by the RL model compared to the control condition. Additionally, participants reported exercising at a higher intensity during sessions generated using the RL model (RL condition: mean intensity 5.82, 95% CI 5.59‐6.05 and baseline condition: mean intensity 5.19, 95% CI 4.97‐5.41).

### Comparison to Prior Work

Our findings have import practical implications. Despite the well-known benefits of regular physical activity, many individuals struggle to stick to an exercise routine over the long term [43,44]. This is often due to a lack of motivation or a mismatch between the exercise program and the individual’s personal preferences and abilities [45-47]. RL-generated sessions, however, make it possible to personalize exercise programs, tailoring them to an individual’s unique preferences, abilities, and goals. This could potentially improve motivation and adherence to the exercise routine, ultimately leading to better health outcomes.

In particular, the ubiquity of smartphones means that app-based exercise prescription, enabled by RL algorithms, has the potential to greatly improve public health by increasing motivation and adherence to exercise routines. RL is a type of artificial intelligence that involves training a model to make decisions that maximize a reward. This is typically done through a process of trial and error, where the model receives a reward for taking certain actions and a penalty for others. Over time, the model learns to make the decisions that are most likely to result in the maximum reward. The process of RL is similar to how animals and humans learn to perform tasks, and it has been used to solve a wide range of problems in fields such as robotics, gaming, and finance [48,49]. The key advantage of RL is its ability to learn from experience and adapt to new situations without needing to be explicitly programmed. This makes it an effective approach for solving complex, dynamic problems such as how to devise an exercise program.

The use of ML methods, including RL, to generate is a burgeoning research area. Previous research has demonstrated the effectiveness of using ML for tailoring interventions in these apps. For instance, Aguilera et al [50] observed a significant improvement in step count among individuals with diabetes and depression over the short term when ML was used to tailor SMS text messages within a self-management app. Additionally, RL has been successfully applied to enhance adherence to exercise in patients with diabetes through the distribution of individualized service messages that encourage physical activity [51]. The application of ML has even shown promise in the general population, with personalized daily step goals leading to increased physical activity compared to static step goals [52].

Our trial is the first known study to use an RL model specifically for personalizing exercise sessions and assessing its impact on participant satisfaction. While prior studies have used RL to promote exercise adherence through personalized messaging, the outcomes were mostly centered around physical activity measures such as step count [52,53]. Although our study and these previous studies differ in design and primary outcomes, a common thread can be observed regarding the potential of RL to encourage physical activity and exercise, despite the challenges inherent in smartphone app delivery. Furthermore, there is a relative scarcity of evidence concerning the use of RL specifically for exercise, as opposed to physical activity. RL has found utility in wider health care contexts, such as individualizing the difficulty level in a virtual reality rehabilitation game [54], aiding in weight loss maintenance [55], and even selecting and delivering drugs [56-58].

These studies, alongside the findings from our trial, reinforce the emerging potential of RL in health-related behaviors and outcomes. The results of this crossover trial contribute to our understanding of the potential for adaptive exercise interventions to improve adherence to an exercise intervention. Our hypothesis that users would be more satisfied with sessions generated by the RL system was confirmed. The effect size, calculated as the mean difference normalized by the SD (Cohen *d*), was approximately 0.24. This represents a small to moderate effect size, suggesting that while the RL model led to higher satisfaction, the magnitude of this difference, though statistically significant, might not be substantial in practical terms. These findings underscore the importance of considering both statistical and practical significance in evaluating the efficacy of health interventions. The practical implications suggest that while the RL model enhances satisfaction, efforts to refine the model to maximize its impact on user engagement and satisfaction should continue. Future research should focus on identifying and integrating additional predictors of satisfaction into the RL model, potentially increasing the effect size and thereby the practical impact of the technology. This research could also inform the development of RL systems for exercise prescription in patient populations (such as those with cardiovascular or metabolic diseases). This could lead to better health outcomes and reduced health care costs, as regular physical activity and exercise are known to prevent a wide range of chronic diseases [59]. Additionally, app-based exercise prescription has the potential to be more accessible and convenient than traditional forms of exercise prescription, making it easier for individuals to incorporate physical activity into their daily lives. On this basis, RL in smartphone app–based exercise prescription has the potential to greatly benefit public health; this study is the first step in setting the empirical foundation for follow-on research to investigate and establish the potential of RL-based exercise prescription in larger and more diverse populations.

Typically, in exercise science research investigating the use of mobile technologies to deliver the exercise intervention, participants are randomized to either an intervention group or a control group [60,61]. These groups are subsequently monitored for a predefined period as they use the app and are measured at the end of the intervention period to determine the health benefits of the exercise intervention, for example, based on a decrease in body weight or BMI, an increase in fat-free mass, or alterations in certain blood biomarkers [62,63]. However, the health benefits of exercise in diverse population groups are now well established. This is one of the primary reasons why we adopted a session-focused, “back and forth” crossover design (which was more robust to reducing levels of participation over the course of the trial), with satisfaction as our primary outcome. By cycling between each intervention condition on a weekly basis, participants acted as their own controls and ensured that we have a representative dataset for both the RL and control conditions. Rather than user-associated outcomes such as body weight at the start and conclusion of the intervention period being the primary experimental end point then, we focused on session-level outcomes like satisfaction and RPE. Specifically, each exercise session was characterized by the aggregated statistics of satisfaction and RPE for our entire cohort. This allowed us to build a profile for each session and evaluate how profiles are altered for each experimental condition under which the sessions are completed (and also to provide continuous input for the RL system).

However, despite its novelty and the strength of its methodological design, this study is not without limitations. Ours was a relatively small sample of homogenous participants; therefore, our findings may not be generalizable to other populations. It is also important to note the potential for selection bias in our recruitment strategy. Participants were recruited through word of mouth and social media, which might not provide a fully representative sample of the target population. This approach could attract individuals who are more technologically savvy, potentially more health-conscious, or with specific demographic characteristics that are overrepresented in social media networks. Consequently, our findings might not be generalizable to all segments of the population. While the crossover study design was statistically efficient, it meant that we were unable to compare 2 separate groups in the long term for our primary and secondary outcomes. It was also not possible to follow a double-blind design, as researchers needed to be aware of participants’ group allocation throughout the intervention period to manage follow-up and ensure that participants did not have any issues using the app; the researchers and technical personnel had to monitor our server for troubleshooting throughout the trial. Finally, we observed that participants completed an average of 9 sessions over the 12-week trial period, equating to less than 1 session per week. This level of engagement is notably lower than the intended 3 sessions per week per participant outlined in the study protocol. Several factors could contribute to this lower-than-expected adherence rate, including potential barriers faced by participants such as time constraints, lack of motivation, or the possibility that the app did not fully engage users as anticipated. Understanding and addressing these factors are crucial for the future development of app-based exercise prescriptions, as enhanced user engagement is key to achieving the health benefits of regular physical activity. The insights gained could also inform the broader field of digital health interventions aimed at improving lifestyle behaviors.

### Conclusions

This randomized crossover trial evaluated the utility of a type of artificial intelligence called RL for devising an app-based system for exercise prescription. Our findings demonstrated that exercise sessions generated using RL were associated with higher satisfaction. Additionally, our participants completed sessions in the RL condition at a higher intensity than in the control condition. Taken together, our results suggest that there is significant scope for using RL algorithms to train a model to recommend exercises that are tailored to an individual’s unique goals, preferences, and abilities. Further research should investigate the potential for exercise prescription using RL to empower the general population to be healthier by providing personalized and engaging exercise programs that are tailored to an individual’s unique goals, preferences, and abilities. Such an approach may help individuals take control of their own health and fitness and provide them with the tools and support they need to maintain a regular exercise routine. By making exercise more accessible, engaging, and personalized, RL has the potential to improve public health by encouraging more individuals to incorporate physical activity into their daily lives.

## Supplementary material

10.2196/49443 Checklist 1 CONSORT-EHEALTH (Consolidated Standards of Reporting Trials of Electronic and Mobile Health Applications and Online Telehealth) checklist (version 1.6.1).

## Abbreviations

GEE: generalized estimating equation
ML: machine learning
PACES-8: 8-item version of the Physical Activity Enjoyment Scale
RL: reinforcement learning
RPE: rate of perceived exertion
SPIRIT: Standard Protocol Items: Recommendations for Interventional Trials

## References

[1] Pedersen BK, Saltin B (2015). Exercise as medicine—evidence for prescribing exercise as therapy in 26 different chronic diseases. Scand Med Sci Sports.

[2] Lavie CJ, O’Keefe JH, Sallis RE (2015). Exercise and the heart—the harm of too little and too much. Curr Sports Med Rep.

[3] Lavie CJ, Ozemek C, Carbone S, Katzmarzyk PT, Blair SN (2019). Sedentary behavior, exercise, and cardiovascular health. Circ Res.

[4] Ross R, Blair SN, Arena R (2016). Importance of assessing cardiorespiratory fitness in clinical practice: a case for fitness as a clinical vital sign: a scientific statement from the American Heart Association. Circulation.

[5] Blair SN, Sallis RE, Hutber A, Archer E (2012). Exercise therapy—the public health message. Scand Med Sci Sports.

[6] Thomas MM, Phongsavan P, McGill B, O’Hara BJ, Bauman AE (2018). A review of the impact of physical activity mass media campaigns on low compared to high socioeconomic groups. Health Educ Res.

[7] Booth FW, Roberts CK, Laye MJ (2012). Lack of exercise is a major cause of chronic diseases. Compr Physiol.

[8] Arocha Rodulfo JI (2019). Sedentarismo, la enfermedad del siglo xxi. Clín Invest Arterioscl.

[9] Johnston CA, Moreno JP, Hernandez DC (2019). Levels of adherence needed to achieve significant weight loss. Int J Obes.

[10] Beaudry R, Kruger C, Liang Y, Parliament M, Haykowsky M, McNeely M (2015). Effect of supervised exercise on aerobic capacity in cancer survivors: adherence and workload predict variance in effect. WJMA.

[11] Hawley-Hague H, Horne M, Skelton DA, Todd C (2016). Review of how we should define (and measure) adherence in studies examining older adults’ participation in exercise classes. BMJ Open.

[12] Slade SC, Dionne CE, Underwood M, Buchbinder R (2016). Consensus on Exercise Reporting Template (CERT): explanation and elaboration statement. Br J Sports Med.

[13] Lemstra M, Bird Y, Nwankwo C, Rogers M, Moraros J (2016). Weight loss intervention adherence and factors promoting adherence: a meta-analysis. Patient Prefer Adherence.

[14] Sirur R, Richardson J, Wishart L, Hanna S (2009). The role of theory in increasing adherence to prescribed practice. Physiother Can.

[15] Lai B, Young HJ, Bickel CS, Motl RW, Rimmer JH (2017). Current trends in exercise intervention research, technology, and behavioral change strategies for people with disabilities. Am J Phys Med Rehabil.

[16] Collado-Mateo D, Lavín-Pérez AM, Peñacoba C (2021). Key factors associated with adherence to physical exercise in patients with chronic diseases and older adults: an umbrella review. Int J Environ Res Public Health.

[17] Bhat AA, DeWalt DA, Zimmer CR, Fried BJ, Callahan LF (2010). The role of helplessness, outcome expectation for exercise and literacy in predicting disability and symptoms in older adults with arthritis. Patient Educ Couns.

[18] Dewalt DA, Berkman ND, Sheridan S, Lohr KN, Pignone MP (2004). Literacy and health outcomes: a systematic review of the literature. J Gen Intern Med.

[19] Keogh A, Argent R, Doherty C (2024). Breaking down the digital fortress: the unseen challenges in healthcare technology—lessons learned from 10 years of research. Sensors (Basel).

[20] (2022). Smartphone-penetration-worldwide-by-country. Statista.

[21] Althoff T, Sosič R, Hicks JL, King AC, Delp SL, Leskovec J (2017). Large-scale physical activity data reveal worldwide activity inequality. Nature New Biol.

[22] Case MA, Burwick HA, Volpp KG, Patel MS (2015). Accuracy of smartphone applications and wearable devices for tracking physical activity data. JAMA.

[23] Hekler EB, Buman MP, Grieco L (2015). Validation of physical activity tracking via android smartphones compared to ActiGraph accelerometer: laboratory-based and free-living validation studies. JMIR Mhealth Uhealth.

[24] Kostopoulos K, Chouvarda I, Koutkias V, Kokonozi A, van Gils M, Maglaveras N An ontology-based framework aiming to support personalized exercise prescription: application in cardiac rehabilitation.

[25] Lofaro D, Groccia MC, Guido R, Conforti D, Caroleo S, Fragomeni G Machine learning approaches for supporting patient:specific cardiac rehabilitation programs.

[26] Lo CL, Tseng HT (2017). Predicting rehabilitation treatment helpfulness to stroke patients: a supervised learning approach. Artif Intell Res.

[27] Hansen D, Dendale P, Coninx K (2017). The European Association of Preventive Cardiology Exercise Prescription in Everyday Practice and Rehabilitative Training (EXPERT) tool: a digital training and decision support system for optimized exercise prescription in cardiovascular disease. Concept, definitions and construction methodology. Eur J Prev Cardiol.

[28] Azar KMJ, Lesser LI, Laing BY (2013). Mobile applications for weight management: theory-based content analysis. Am J Prev Med.

[29] Bardus M, van Beurden SB, Smith JR, Abraham C (2016). A review and content analysis of engagement, functionality, aesthetics, information quality, and change techniques in the most popular commercial apps for weight management. Int J Behav Nutr Phys Act.

[30] Mercer K, Li M, Giangregorio L, Burns C, Grindrod K (2016). Behavior change techniques present in wearable activity trackers: a critical analysis. JMIR Mhealth Uhealth.

[31] Breslin J, Vickey T, Williams A (2013). Fitness—there’s an app for that: review of mobile fitness apps. Int J Sport Soc.

[32] McArthur LH, Raedeke TD (2009). Race and sex differences in college student physical activity correlates. Am J Health Behav.

[33] Raedeke TD (2007). The relationship between enjoyment and affective responses to exercise. J Appl Sport Psychol.

[34] Chan AW, Tetzlaff JM, Altman DG (2013). SPIRIT 2013 statement: defining standard protocol items for clinical trials. Ann Intern Med.

[35] Kim C, Kim SH, Suh MR (2022). Accuracy and validity of commercial smart bands for heart rate measurements during cardiopulmonary exercise test. Ann Rehabil Med.

[36] Spinsante S, Porfiri S, Scalise L Accuracy of heart rate measurements by a smartwatch in low intensity activities.

[37] Xie J, Wen D, Liang L, Jia Y, Gao L, Lei J (2018). Evaluating the validity of current mainstream wearable devices in fitness tracking under various physical activities: comparative study. JMIR Mhealth Uhealth.

[38] Tragos E, O’Reilly-Morgan D, Geraci J Keeping people active and healthy at home using a reinforcement learning-based fitness recommendation framework. https://www.ijcai.org/proceedings/2023.

[39] Terwee CB, Bot SDM, de Boer MR (2007). Quality criteria were proposed for measurement properties of health status questionnaires. J Clin Epidemiol.

[40] Bird ML, Clark B, Millar J, Whetton S, Smith S (2015). Exposure to “exergames” increases older adults’ perception of the usefulness of technology for improving health and physical activity: a pilot study. JMIR Serious Games.

[41] Forsyth L, Bonacci J, Childs C (2022). A pilot randomised control trial of the efficacy of stability-based training with visualisation for people with chronic ankle instability. Med Biol Eng Comput.

[42] Taylor ME, Close JCT, Lord SR (2020). Pilot feasibility study of a home-based fall prevention exercise program (StandingTall) delivered through a tablet computer (iPad) in older people with dementia. Australas J Ageing.

[43] Li CL, Lai YC, Tseng CH, Lin JD, Chang HY (2010). A population study on the association between leisure time physical activity and self-rated health among diabetics in Taiwan. BMC Public Health.

[44] Artistico D, Pinto AM, Douek J, Black J, Pezzuti L (2013). The value of removing daily obstacles via everyday problem-solving theory: developing an applied novel procedure to increase self-efficacy for exercise. Front Psychol.

[45] Chard S (2017). Qualitative perspectives on aquatic exercise initiation and satisfaction among persons with multiple sclerosis. Disabil Rehabil.

[46] Sun T, Xu Y, Xie H, Ma Z, Wang Y (2021). Intelligent personalized exercise prescription based on an eHealth promotion system to improve health outcomes of middle-aged and older adult community dwellers: pretest-posttest study. J Med Internet Res.

[47] Sutin AR, Stephan Y, Luchetti M, Artese A, Oshio A, Terracciano A (2016). The five-factor model of personality and physical inactivity: a meta-analysis of 16 samples. J Res Pers.

[48] Abdulhameed SA, Lupenko S (2022). Potentials of reinforcement learning in contemporary scenarios. Sci J TNTU.

[49] Charpentier A, Élie R, Remlinger C (2021). Reinforcement learning in economics and finance. Comput Econ.

[50] Aguilera A, Figueroa CA, Hernandez-Ramos R (2020). mHealth app using machine learning to increase physical activity in diabetes and depression: clinical trial protocol for the DIAMANTE Study. BMJ Open.

[51] Zhou M, Fukuoka Y, Mintz Y (2018). Evaluating machine learning-based automated personalized daily step goals delivered through a mobile phone app: randomized controlled trial. JMIR Mhealth Uhealth.

[52] Yom-Tov E, Feraru G, Kozdoba M, Mannor S, Tennenholtz M, Hochberg I (2017). Encouraging physical activity in patients with diabetes: intervention using a reinforcement learning system. J Med Internet Res.

[53] Zhou M, Mintz Y, Fukuoka Y (2018). Personalizing mobile fitness apps using reinforcement learning. CEUR Workshop Proc.

[54] Ávila-Sansores S, Orihuela-Espina F, Enrique-Sucar L (2013). Converging Clinical and Engineering Research on Neurorehabilitation.

[55] Forman EM, Kerrigan SG, Butryn ML (2019). Can the artificial intelligence technique of reinforcement learning use continuously-monitored digital data to optimize treatment for weight loss?. J Behav Med.

[56] Parbhoo S (2014). A Reinforcement Learning Design for HIV Clinical Trials [PhD thesis]. https://wiredspace.wits.ac.za/server/api/core/bitstreams/c45179b7-e1e5-47cd-876a-665ed1f3019f/content.

[57] Daskalaki E, Diem P, Mougiakakou SG (2013). Personalized tuning of a reinforcement learning control algorithm for glucose regulation. Annu Int Conf IEEE Eng Med Biol Soc.

[58] Escandell-Montero P, Chermisi M, Martínez-Martínez JM (2014). Optimization of anemia treatment in hemodialysis patients via reinforcement learning. Artif Intell Med.

[59] (2023). Physical activity—key facts. World Health Organization.

[60] Hurling R, Catt M, Boni MD (2007). Using internet and mobile phone technology to deliver an automated physical activity program: randomized controlled trial. J Med Internet Res.

[61] Allen JK, Stephens J, Dennison Himmelfarb CR, Stewart KJ, Hauck S (2013). Randomized controlled pilot study testing use of smartphone technology for obesity treatment. J Obes.

[62] Spring B, Pellegrini CA, Pfammatter A (2017). Effects of an abbreviated obesity intervention supported by mobile technology: the ENGAGED randomized clinical trial. Obesity (Silver Spring).

[63] Lahtio H, Rintala A, Immonen J, Sjögren T (2022). The effectiveness of physical activity-promoting web- and mobile-based distance weight loss interventions on body composition in rehabilitation settings: systematic review, meta-analysis, and meta-regression analysis. J Med Internet Res.

