# Supplementary material for: An Evaluation of the Effect of App-Based Exercise Prescription Using Reinforcement Learning on Satisfaction and Exercise Intensity: Randomized Crossover Trial
Source: JMIR Mhealth Uhealth. 2024 Nov 26;12:e49443. doi: 10.2196/49443 (PMC11612604; doi:10.2196/49443)
Supplement: Checklist 1 [file mhealth-v12-e49443-s001.pdf]

CONSORT-EHEALTH (V 1.6.1) - Submission/Publication Form

The CONSORT-EHEALTH checklist is intended for authors of randomized trials evaluating web-based and Internet-based applications/interventions, including mobile interventions, electronic games (incl multiplayer games), social media, certain telehealth applications, and other interactive and/or networked electronic applications. Some of the items (e.g. all subitems under item 5 - description of the intervention) may also be applicable for other study designs.

The goal of the CONSORT EHEALTH checklist and guideline is to be
a) a guide for reporting for authors of RCTs,
b) to form a basis for appraisal of an ehealth trial (in terms of validity)

CONSORT-EHEALTH items/subitems are MANDATORY reporting items for studies published in the Journal of Medical Internet Research and other journals / scientific societies endorsing the checklist.

Items numbered 1., 2., 3., 4a., 4b etc are original CONSORT or CONSORT-NPT (non-pharmacologic treatment) items.
Items with Roman numerals (i., ii, iii, iv etc.) are CONSORT-EHEALTH extensions/clarifications.

As the CONSORT-EHEALTH checklist is still considered in a formative stage, we would ask that you also RATE ON A SCALE OF 1-5 how important/useful you feel each item is FOR THE PURPOSE OF THE CHECKLIST and reporting guideline (optional).

Mandatory reporting items are marked with a red *.
In the textboxes, either copy & paste the relevant sections from your manuscript into this form - please include any quotes from your manuscript in QUOTATION MARKS,
or answer directly by providing additional information not in the manuscript, or elaborating on why the item was not relevant for this study.

YOUR ANSWERS WILL BE PUBLISHED AS A SUPPLEMENTARY FILE TO YOUR PUBLICATION IN JMIR AND ARE CONSIDERED PART OF YOUR PUBLICATION (IF ACCEPTED).
Please fill in these questions diligently. Information will not be copyedited, so please use proper spelling and grammar, use correct capitalization, and avoid abbreviations.

DO NOT FORGET TO SAVE AS PDF _AND_ CLICK THE SUBMIT BUTTON SO YOUR ANSWERS ARE IN OUR DATABASE !!!

Citation Suggestion (if you append the pdf as Appendix we suggest to cite this paper in the caption):
Eysenbach G, CONSORT-EHEALTH Group
CONSORT-EHEALTH: Improving and Standardizing Evaluation Reports of Web-based and Mobile Health Interventions
J Med Internet Res 2011;13(4):e126
URL: http://www.jmir.org/2011/4/e126/
doi: 10.2196/jmir.1923
PMID: 22209829

cailbhedoherty@gmail.com Switch account
Not shared
Draft not saved

* Indicates required question

Your name *
First Last

Cailbhe Doherty

Primary Affiliation (short), City, Country *
University of Toronto, Toronto, Canada

University College Dublin

Your e-mail address *
abc@gmail.com

cailbhe.doherty@insight-centre.org

Title of your manuscript *
Provide the (draft) title of your manuscript.

An evaluation of the Effect of App-Based Exercise Prescription Using RL on Satisfaction and Exercise Intensity: Randomized Crossover Trial

Name of your App/Software/Intervention *
If there is a short and a long/alternate name, write the short name first and add the long name in brackets.

i80 BPM

Evaluated Version (if any)
e.g. "V1", "Release 2017-03-01", "Version 2.0.27913"

N/A

Your answer must have a minimum of 5 characters.

Language(s) *
What language is the intervention/app in? If multiple languages are available, separate by comma (e.g. "English, French")

English, Korean

URL of your Intervention Website or App
e.g. a direct link to the mobile app on app in appstore (itunes, Google Play), or URL of the website. If the intervention is a DVD or hardware, you can also link to an Amazon page.

The app is not publicly available as it was still in development during the study.

Must be a valid URL

URL of an image/screenshot (optional)

N/A

Accessibility *
Can an enduser access the intervention presently?

- ○ access is free and open
- ◉ access only for special usergroups, not open
- ○ access is open to everyone, but requires payment/subscription/in-app purchases
- ○ app/intervention no longer accessible
- ○ Other:

Primary Medical Indication/Disease/Condition *
e.g. "Stress", "Diabetes", or define the target group in brackets after the condition, e.g. "Autism (Parents of children with)", "Alzheimers (Informal Caregivers of)"

Sedentary lifestyle, exercise adherence

Primary Outcomes measured in trial *
comma-separated list of primary outcomes reported in the trial

User satisfaction (PACES-8 scale), exercise int

Secondary/other outcomes
Are there any other outcomes the intervention is expected to affect?

Heart rate (measured via wearable device), adherence rates

Recommended "Dose" *
What do the instructions for users say on how often the app should be used?

- ○ Approximately Daily
- ◉ Approximately Weekly
- ○ Approximately Monthly
- ○ Approximately Yearly
- ○ "as needed"
- ○ Other:

Approx. Percentage of Users (starters) still using the app as recommended after 3 months *

- ○ unknown / not evaluated
- ○ 0-10%
- ○ 11-20%
- ○ 21-30%
- ○ 31-40%
- ○ 41-50%
- ○ 51-60%
- ◉ 61-70%
- ○ 71%-80%
- ○ 81-90%
- ○ 91-100%
- ○ Other:

Overall, was the app/intervention effective? *

- ○ yes: all primary outcomes were significantly better in intervention group vs control
- ◉ partly: SOME primary outcomes were significantly better in intervention group vs control
- ○ no statistically significant difference between control and intervention
- ○ potentially harmful: control was significantly better than intervention in one or more outcomes
- ○ inconclusive: more research is needed
- ○ Other:

Article Preparation Status/Stage *
At which stage in your article preparation are you currently (at the time you fill in this form)

- ○ not submitted yet - in early draft status
- ○ not submitted yet - in late draft status, just before submission
- ○ submitted to a journal but not reviewed yet
- ○ submitted to a journal and after receiving initial reviewer comments
- ◉ submitted to a journal and accepted, but not published yet
- ○ published
- ○ Other:

Journal *
If you already know where you will submit this paper (or if it is already submitted), please provide the journal name (if it is not JMIR, provide the journal name under "other")

- ○ not submitted yet / unclear where I will submit this
- ○ Journal of Medical Internet Research (JMIR)
- ◉ JMIR mHealth and UHealth
- ○ JMIR Serious Games
- ○ JMIR Mental Health
- ○ JMIR Public Health
- ○ JMIR Formative Research
- ○ Other JMIR sister journal
- ○ Other:

Is this a full powered effectiveness trial or a pilot/feasibility trial? *

- ○ Pilot/feasibility
- ◉ Fully powered

Manuscript tracking number *
If this is a JMIR submission, please provide the manuscript tracking number under "other" (The ms tracking number can be found in the submission acknowledgement email, or when you login as author in JMIR. If the paper is already published in JMIR, then the ms tracking number is the four-digit number at the end of the DOI, to be found at the bottom of each published article in JMIR)

- ○ no ms number (yet) / not (yet) submitted to / published in JMIR
- ◉ Other: JMIR mHealth and uHealth Manuscript #49443

TITLE AND ABSTRACT

1a) TITLE: Identification as a randomized trial in the title

1a) Does your paper address CONSORT item 1a? *
I.e does the title contain the phrase "Randomized Controlled Trial"? (if not, explain the reason under "other")

- ◉ yes
- ○ Other:

1a-i) Identify the mode of delivery in the title
Identify the mode of delivery. Preferably use "web-based" and/or "mobile" and/or "electronic game" in the title. Avoid ambiguous terms like "online", "virtual", "interactive". Use "Internet-based" only if Intervention includes non-web-based Internet components (e.g. email), use "computer-based" or "electronic" only if offline products are used. Use "virtual" only in the context of "virtual reality" (3-D worlds). Use "online" only in the context of "online support groups". Complement or substitute product names with broader terms for the class of products (such as "mobile" or "smart phone" instead of "iphone"), especially if the application runs on different platforms.

subitem not at all important 1 ○ 2 ○ 3 ○ 4 ○ 5 ◉ essential

Clear selection

Does your paper address subitem 1a-i? *
Copy and paste relevant sections from manuscript title (include quotes in quotation marks "like this" to indicate direct quotes from your manuscript), or elaborate on this item by providing additional information not in the ms, or briefly explain why the item is not applicable/relevant for your study

The title mentions the mode of delivery: "App-Based Exercise Prescription."

1a-ii) Non-web-based components or important co-interventions in title
Mention non-web-based components or important co-interventions in title, if any (e.g., "with telephone support").

subitem not at all important 1 ◉ 2 ○ 3 ○ 4 ○ 5 ○ essential

Clear selection

Does your paper address subitem 1a-ii?
Copy and paste relevant sections from manuscript title (include quotes in quotation marks "like this" to indicate direct quotes from your manuscript), or elaborate on this item by providing additional information not in the ms, or briefly explain why the item is not applicable/relevant for your study

Not applicable for this study.

1a-iii) Primary condition or target group in the title
Mention primary condition or target group in the title, if any (e.g., "for children with Type I Diabetes") Example: A Web-based and Mobile Intervention with Telephone Support for Children with Type I Diabetes: Randomized Controlled Trial

subitem not at all important 1 ○ 2 ○ 3 ○ 4 ○ 5 ◉ essential

Clear selection

Does your paper address subitem 1a-iii? *
Copy and paste relevant sections from manuscript title (include quotes in quotation marks "like this" to indicate direct quotes from your manuscript), or elaborate on this item by providing additional information not in the ms, or briefly explain why the item is not applicable/relevant for your study

The primary focus of the intervention is on exercise prescription, and this is addressed in the title.

1b) ABSTRACT: Structured summary of trial design, methods, results, and conclusions
NPT extension: Description of experimental treatment, comparator, care providers, centers, and blinding status.

1b-i) Key features/functionalities/components of the intervention and comparator in the METHODS section of the ABSTRACT
Mention key features/functionalities/components of the intervention and comparator in the abstract. If possible, also mention theories and principles used for designing the site. Keep in mind the needs of systematic reviewers and indexers by including important synonyms. (Note: Only report in the abstract what the main paper is reporting. If this information is missing from the main body of text, consider adding it)

subitem not at all important 1 ○ 2 ○ 3 ○ 4 ○ 5 ◉ essential

Clear selection

Does your paper address subitem 1b-i? *
Copy and paste relevant sections from the manuscript abstract (include quotes in quotation marks "like this" to indicate direct quotes from your manuscript), or elaborate on this item by providing additional information not in the ms, or briefly explain why the item is not applicable/relevant for your study

The abstract includes a structured summary with the trial design, methods, key results, and conclusions.
Quote: "This study evaluated the impact of personalised exercise sessions using reinforcement learning on user satisfaction and exercise intensity in a 12-week randomised crossover trial."

1b-ii) Level of human involvement in the METHODS section of the ABSTRACT
Clarify the level of human involvement in the abstract, e.g., use phrases like "fully automated" vs. "therapist/nurse/care provider/physician-assisted" (mention number and expertise of providers involved, if any). (Note: Only report in the abstract what the main paper is reporting. If this information is missing from the main body of text, consider adding it)

subitem not at all important 1 ○ 2 ○ 3 ◉ 4 ○ 5 ○ essential

Clear selection

Does your paper address subitem 1b-ii?
Copy and paste relevant sections from the manuscript abstract (include quotes in quotation marks "like this" to indicate direct quotes from your manuscript), or elaborate on this item by providing additional information not in the ms, or briefly explain why the item is not applicable/relevant for your study

The app intervention was fully automated. Participants interacted directly with the app, and no human support was involved during the exercise sessions.

1b-iii) Open vs. closed, web-based (self-assessment) vs. face-to-face assessments in the METHODS section of the ABSTRACT
Mention how participants were recruited (online vs. offline), e.g., from an open access website or from a clinic or a closed online user group (closed usergroup trial), and clarify if this was a purely web-based trial, or there were face-to-face components (as part of the intervention or for assessment). Clearly say if outcomes were self-assessed through questionnaires (as common in web-based trials). Note: In traditional offline trials, an open trial (open-label trial) is a type of clinical trial in which both the researchers and participants know which treatment is being administered. To avoid confusion, use "blinded" or "unblinded" to indicated the level of blinding instead of "open", as "open" in web-based trials usually refers to "open access" (i.e. participants can self-enrol). (Note: Only report in the abstract what the main paper is reporting. If this information is missing from the main body of text, consider adding it)

subitem not at all important 1 ○ 2 ○ 3 ◉ 4 ○ 5 ○ essential

Clear selection

Does your paper address subitem 1b-iii?
Copy and paste relevant sections from the manuscript abstract (include quotes in quotation marks "like this" to indicate direct quotes from your manuscript), or elaborate on this item by providing additional information not in the ms, or briefly explain why the item is not applicable/relevant for your study

Participants were recruited via offline methods, and outcomes were self-assessed through the app. There were no face-to-face components.
Quote: "Participants self-reported satisfaction and intensity after each session through the app."

1b-iv) RESULTS section in abstract must contain use data
Report number of participants enrolled/assessed in each group, the use/uptake of the intervention (e.g., attrition/adherence metrics, use over time, number of logins etc.), in addition to primary/secondary outcomes. (Note: Only report in the abstract what the main paper is reporting. If this information is missing from the main body of text, consider adding it)

subitem not at all important 1 ○ 2 ○ 3 ○ 4 ◉ 5 ○ essential

Clear selection

Does your paper address subitem 1b-iv?
Copy and paste relevant sections from the manuscript abstract (include quotes in quotation marks "like this" to indicate direct quotes from your manuscript), or elaborate on this item by providing additional information not in the ms, or briefly explain why the item is not applicable/relevant for your study

The abstract includes the number of participants and summarises usage and outcome data.
Quote: "62 participants completed at least one session, with 559 sessions in total."

1b-v) CONCLUSIONS/DISCUSSION in abstract for negative trials
Conclusions/Discussions in abstract for negative trials: Discuss the primary outcome - if the trial is negative (primary outcome not changed), and the intervention was not used, discuss whether negative results are attributable to lack of uptake and discuss reasons. (Note: Only report in the abstract what the main paper is reporting. If this information is missing from the main body of text, consider adding it)

subitem not at all important 1 ○ 2 ◉ 3 ○ 4 ○ 5 ○ essential

Clear selection

Does your paper address subitem 1b-v?
Copy and paste relevant sections from the manuscript abstract (include quotes in quotation marks "like this" to indicate direct quotes from your manuscript), or elaborate on this item by providing additional information not in the ms, or briefly explain why the item is not applicable/relevant for your study

Not applicable—this study had positive results.

INTRODUCTION

2a) In INTRODUCTION: Scientific background and explanation of rationale

2a-i) Problem and the type of system/solution
Describe the problem and the type of system/solution that is object of the study: intended as stand-alone intervention vs. incorporated in broader health care program? Intended for a particular patient population? Goals of the intervention, e.g., being more cost-effective to other interventions, replace or complement other solutions? (Note: Details about the intervention are provided in "Methods" under 5)

subitem not at all important 1 ○ 2 ○ 3 ○ 4 ◉ 5 ○ essential

Clear selection

Does your paper address subitem 2a-i? *
Copy and paste relevant sections from the manuscript (include quotes in quotation marks "like this" to indicate direct quotes from your manuscript), or elaborate on this item by providing additional information not in the ms, or briefly explain why the item is not applicable/relevant for your study

Yes. The problem of low exercise adherence is addressed, and the system/solution being evaluated is a smartphone app incorporating reinforcement learning (RL) to tailor exercise sessions.
Quote: "Despite this, inactivity (and the increased risk of morbidity and mortality associated with it) remains a significant public health concern. [...] Many theories and models have been proposed to explain why adherence to exercise is suboptimal."

2a-ii) Scientific background, rationale: What is known about the (type of) system
Scientific background, rationale: What is known about the (type of) system that is the object of the study (be sure to discuss the use of similar systems for other conditions/diagnoses, if appropiate), motivation for the study, i.e. what are the reasons for and what is the context for this specific study, from which stakeholder viewpoint is the study performed, potential impact of findings [2]. Briefly justify the choice of the comparator.

subitem not at all important 1 ○ 2 ○ 3 ○ 4 ◉ 5 ○ essential

Clear selection

Does your paper address subitem 2a-ii? *
Copy and paste relevant sections from the manuscript (include quotes in quotation marks "like this" to indicate direct quotes from your manuscript), or elaborate on this item by providing additional information not in the ms, or briefly explain why the item is not applicable/relevant for your study

Yes. The introduction provides the scientific rationale behind the app, explaining both the health benefits of exercise and the potential for technology to deliver personalised exercise programs. It discusses the background of mobile technologies and the benefits of using reinforcement learning for exercise prescription.
Quote: "Mobile technologies (e.g., smartphone apps and wearable activity trackers) are a cost-effective, scalable way of delivering exercise programs to users that incorporate these factors."
It further highlights the gap in current research, as comprehensive studies evaluating fully computerised, app-based exercise prescription remain limited.
Quote: "Although several frameworks exist for automating exercise prescription based on user demographics, fitness levels, engagement behaviours, and preferences, comprehensive studies evaluating the effectiveness of fully computerised, app-based exercise prescription remain limited."

2b) In INTRODUCTION: Specific objectives or hypotheses

Does your paper address CONSORT subitem 2b? *
Copy and paste relevant sections from the manuscript (include quotes in quotation marks "like this" to indicate direct quotes from your manuscript), or elaborate on this item by providing additional information not in the ms, or briefly explain why the item is not applicable/relevant for your study

Yes. The study clearly defines its objectives and hypotheses. The primary objective is to evaluate whether reinforcement learning improves user satisfaction compared to generic exercise sessions, and the secondary objective is to assess exercise intensity through perceived exertion.
Quote: "Our primary hypotheses were that users would report higher satisfaction and demonstrate greater adherence to exercise programs generated using RL, compared with sessions that are generated randomly or using predefined (i.e., non-personalised) templates."

METHODS

3a) Description of trial design (such as parallel, factorial) including allocation ratio

Does your paper address CONSORT subitem 3a? *
Copy and paste relevant sections from the manuscript (include quotes in quotation marks "like this" to indicate direct quotes from your manuscript), or elaborate on this item by providing additional information not in the ms, or briefly explain why the item is not applicable/relevant for your study

Yes, the trial design is described as a 12-week, assessor-blinded, randomized crossover trial. Participants alternated weekly between the intervention (RL-based exercise sessions) and control (generic, non-personalised sessions) conditions.
Quote: "The design was a 12-week, assessor-blinded, randomized crossover trial, with the primary endpoint being user satisfaction after each exercise session."

3b) Important changes to methods after trial commencement (such as eligibility criteria), with reasons

Does your paper address CONSORT subitem 3b? *
Copy and paste relevant sections from the manuscript (include quotes in quotation marks "like this" to indicate direct quotes from your manuscript), or elaborate on this item by providing additional information not in the ms, or briefly explain why the item is not applicable/relevant for your study

No important changes were made to the methods after the trial commenced. The eligibility criteria, trial design, and outcome measures remained consistent throughout the study.
Quote: "The protocol for this study was developed using the Standard Protocol Items: Recommendations for Interventional Trials checklist."

3b-i) Bug fixes, Downtimes, Content Changes
Bug fixes, Downtimes, Content Changes: ehealth systems are often dynamic systems. A description of changes to methods therefore also includes important changes made on the intervention or comparator during the trial (e.g., major bug fixes or changes in the functionality or content) (5-iii) and other "unexpected events" that may have influenced study design such as staff changes, system failures/downtimes, etc. [2].

subitem not at all important 1 ○ 2 ○ 3 ○ 4 ◉ 5 ○ essential

Clear selection

Does your paper address subitem 3b-i?
Copy and paste relevant sections from the manuscript (include quotes in quotation marks "like this" to indicate direct quotes from your manuscript), or elaborate on this item by providing additional information not in the ms, or briefly explain why the item is not applicable/relevant for your study

No major changes in functionality or bug fixes occurred during the trial. The app's development had already integrated user feedback from an earlier feasibility study. The version used during this trial was the final iteration based on those inputs.
Quote: "The version of the app that was used in the current study... integrated all participants' feedback from this initial feasibility trial."

4a) Eligibility criteria for participants

Does your paper address CONSORT subitem 4a? *
Copy and paste relevant sections from the manuscript (include quotes in quotation marks "like this" to indicate direct quotes from your manuscript), or elaborate on this item by providing additional information not in the ms, or briefly explain why the item is not applicable/relevant for your study

Yes, eligibility criteria are clearly defined. Participants were healthy, recreationally active adults aged 18 to 65 years, who had engaged in aerobic activity for 80 minutes at moderate intensity or less, twice per week.
Quote: "Participants were recruited from Dublin, Ireland... In order to be eligible for inclusion, participants were required to be healthy, recreationally active adults."

4a-i) Computer / Internet literacy
Computer / Internet literacy is often an implicit "de facto" eligibility criterion - this should be explicitly clarified.

subitem not at all important 1 ○ 2 ○ 3 ◉ 4 ○ 5 ○ essential

Clear selection

Does your paper address subitem 4a-i?
Copy and paste relevant sections from the manuscript (include quotes in quotation marks "like this" to indicate direct quotes from your manuscript), or elaborate on this item by providing additional information not in the ms, or briefly explain why the item is not applicable/relevant for your study

While not explicitly stated, participants needed sufficient computer and internet literacy to download and use the smartphone app.
Quote: "Participants attended an information session via Zoom, where they were shown how to use the app and its various functions."

4a-ii) Open vs. closed, web-based vs. face-to-face assessments:
Open vs. closed, web-based vs. face-to-face assessments: Mention how participants were recruited (online vs. offline), e.g., from an open access website or from a clinic, and clarify if this was a purely web-based trial, or there were face-to-face components (as part of the intervention or for assessment), i.e., to what degree got the study team to know the participant. In online-only trials, clarify if participants were quasi-anonymous and whether having multiple identities was possible or whether technical or logistical measures (e.g., cookies, email confirmation, phone calls) were used to detect/prevent these.

subitem not at all important 1 ○ 2 ○ 3 ○ 4 ◉ 5 ○ essential

Clear selection

Does your paper address subitem 4a-ii? *
Copy and paste relevant sections from the manuscript (include quotes in quotation marks "like this" to indicate direct quotes from your manuscript), or elaborate on this item by providing additional information not in the ms, or briefly explain why the item is not applicable/relevant for your study

Our trial involved closed assessments and interactions, conducted through online and mobile platforms. There were no face-to-face components, and all sessions were self-reported via the app.

Quote: "Participants self-reported satisfaction and intensity after each session through the app."

4a-iii) Information giving during recruitment
Information given during recruitment. Specify how participants were briefed for recruitment and in the informed consent procedures (e.g., publish the informed consent documentation as appendix, see also item X26), as this information may have an effect on user self-selection, user expectation and may also bias results.

1 2 3 4 5
subitem not at all important ○ ○ ○ ◉ ○ essential
Clear selection

Does your paper address subitem 4a-iii?
Copy and paste relevant sections from the manuscript (include quotes in quotation marks "like this" to indicate direct quotes from your manuscript), or elaborate on this item by providing additional information not in the ms, or briefly explain why the item is not applicable/relevant for your study

Yes, participants were provided detailed information about the study via email and an information session conducted over Zoom.

Quote: "Upon expressing interest, potential participants received detailed information about the trial along with a health screening questionnaire."

4b) Settings and locations where the data were collected

Does your paper address CONSORT subitem 4b? *
Copy and paste relevant sections from the manuscript (include quotes in quotation marks "like this" to indicate direct quotes from your manuscript), or elaborate on this item by providing additional information not in the ms, or briefly explain why the item is not applicable/relevant for your study

Yes, data were collected via the smartphone app, which participants used in their own environments. The recruitment and study took place in Dublin, Ireland, with participants completing the exercises remotely.

Quote: "Participants were recruited from Dublin, Ireland and its environs via word of mouth and social media."

4b-i) Report if outcomes were (self-)assessed through online questionnaires
Clearly report if outcomes were (self-)assessed through online questionnaires (as common in web-based trials) or otherwise.

1 2 3 4 5
subitem not at all important ○ ○ ○ ◉ ○ essential
Clear selection

Does your paper address subitem 4b-i? *
Copy and paste relevant sections from the manuscript (include quotes in quotation marks "like this" to indicate direct quotes from your manuscript), or elaborate on this item by providing additional information not in the ms, or briefly explain why the item is not applicable/relevant for your study

Yes, the outcomes were self-assessed through the app after each exercise session. User satisfaction and perceived exertion were measured using questionnaires within the app, with no in-person assessments involved.

Quote: "Our primary outcome, user satisfaction, was determined after each session and was collected via the smartphone app. Satisfaction was measured using an abbreviated 8-item version of the Physical Activity Enjoyment Scale (PACES)."

Quote: "Our secondary outcome was perceived exertion, which was measured using the Borg scale, which was administered via the smartphone app at the end of each session."

4b-ii) Report how institutional affiliations are displayed
Report how institutional affiliations are displayed to potential participants [on ehealth media], as affiliations with prestigious hospitals or universities may affect volunteer rates, use, and reactions with regards to an intervention.(Not a required item – describe only if this may bias results)

1 2 3 4 5
subitem not at all important ○ ○ ◉ ○ ○ essential
Clear selection

Does your paper address subitem 4b-ii?
Copy and paste relevant sections from the manuscript (include quotes in quotation marks "like this" to indicate direct quotes from your manuscript), or elaborate on this item by providing additional information not in the ms, or briefly explain why the item is not applicable/relevant for your study

Yes - our affiliations are presented in the title page.

5) The interventions for each group with sufficient details to allow replication, including how and when they were actually administered

5-i) Mention names, credential, affiliations of the developers, sponsors, and owners
Mention names, credential, affiliations of the developers, sponsors, and owners [6] (if authors/evaluators are owners or developer of the software, this needs to be declared in a "Conflict of interest" section or mentioned elsewhere in the manuscript).

1 2 3 4 5
subitem not at all important ○ ○ ◉ ○ ○ essential
Clear selection

Does your paper address subitem 5-i?
Copy and paste relevant sections from the manuscript (include quotes in quotation marks "like this" to indicate direct quotes from your manuscript), or elaborate on this item by providing additional information not in the ms, or briefly explain why the item is not applicable/relevant for your study

We mention the developers and sponsors involved in the study. The i80 BPM app was developed by Samsung Electronics Co., Ltd. The study was conducted at University College Dublin (UCD), and the project team includes researchers affiliated with UCD. The funding source or any external sponsors for the study are not specifically mentioned in this section, but Samsung's role as the developer of the app is clearly stated.

Quote: "Participants were asked to complete three exercise sessions weekly over 12 weeks using the i80 BPM smartphone application, developed by Samsung Electronics Co., Ltd."

Quote: "The UCD Human Research Ethics Committee approved this study (LS-21-34-Tragos-Lawlor)."

5-ii) Describe the history/development process
Describe the history/development process of the application and previous formative evaluations (e.g., focus groups, usability testing), as these will have an impact on adoption/use rates and help with interpreting results.

1 2 3 4 5
subitem not at all important ○ ○ ◉ ○ ○ essential
Clear selection

Does your paper address subitem 5-ii?
Copy and paste relevant sections from the manuscript (include quotes in quotation marks "like this" to indicate direct quotes from your manuscript), or elaborate on this item by providing additional information not in the ms, or briefly explain why the item is not applicable/relevant for your study

Yes, the manuscript provides a description of the app development process, including a prior feasibility study that informed the final version of the app used in this trial. The app was iterated based on participant feedback from the initial feasibility trial, which helped refine its user interface and session structure.
Quote: "During an initial feasibility trial, members of the public worked with us to evaluate the sessions generated by the RL system, the mobile app user interface and were asked to assess the burden and time commitment of the study as part of a user-centred design approach."
Quote: "The version of the app that was used in the current study... integrated all participants' feedback from this initial feasibility trial."

5-iii) Revisions and updating
Revisions and updating. Clearly mention the date and/or version number of the application/intervention (and comparator, if applicable) evaluated, or describe whether the intervention underwent major changes during the evaluation process, or whether the development and/or content was "frozen" during the trial. Describe dynamic components such as news feeds or changing content which may have an impact on the replicability of the intervention (for unexpected events see item 3b).

1 2 3 4 5
subitem not at all important ○ ○ ○ ◉ ○ essential
Clear selection

Does your paper address subitem 5-iii?
Copy and paste relevant sections from the manuscript (include quotes in quotation marks "like this" to indicate direct quotes from your manuscript), or elaborate on this item by providing additional information not in the ms, or briefly explain why the item is not applicable/relevant for your study

Quote: "The version of the app that was used in the current study... integrated all participants' feedback from this initial feasibility trial."

5-iv) Quality assurance methods
Provide information on quality assurance methods to ensure accuracy and quality of information provided [1], if applicable.

1 2 3 4 5
subitem not at all important ○ ○ ○ ◉ ○ essential
Clear selection

Does your paper address subitem 5-iv?
Copy and paste relevant sections from the manuscript (include quotes in quotation marks "like this" to indicate direct quotes from your manuscript), or elaborate on this item by providing additional information not in the ms, or briefly explain why the item is not applicable/relevant for your study

N/A

Your answer must have a minimum of 25 characters.

5-v) Ensure replicability by publishing the source code, and/or providing screenshots/screen-capture video, and/or providing flowcharts of the algorithms used
Ensure replicability by publishing the source code, and/or providing screenshots/screen-capture video, and/or providing flowcharts of the algorithms used. Replicability (i.e., other researchers should in principle be able to replicate the study) is a hallmark of scientific reporting.

1 2 3 4 5
subitem not at all important ○ ○ ○ ◉ ○ essential
Clear selection

Does your paper address subitem 5-v?
Copy and paste relevant sections from the manuscript (include quotes in quotation marks "like this" to indicate direct quotes from your manuscript), or elaborate on this item by providing additional information not in the ms, or briefly explain why the item is not applicable/relevant for your study

No - the app is not open source and represents the industry partner's IP

5-vi) Digital preservation
Digital preservation: Provide the URL of the application, but as the intervention is likely to change or disappear over the course of the years; also make sure the intervention is archived (Internet Archive, webcitation.org, and/or publishing the source code or screenshots/videos alongside the article). As pages behind login screens cannot be archived, consider creating demo pages which are accessible without login.

1 2 3 4 5
subitem not at all important ○ ○ ○ ◉ ○ essential
Clear selection

Does your paper address subitem 5-vi?
Copy and paste relevant sections from the manuscript (include quotes in quotation marks "like this" to indicate direct quotes from your manuscript), or elaborate on this item by providing additional information not in the ms, or briefly explain why the item is not applicable/relevant for your study

N/A see point above concerning IP.

5-vii) Access
Access: Describe how participants accessed the application, in what setting/context, if they had to pay (or were paid) or not, whether they had to be a member of specific group. If known, describe how participants obtained "access to the platform and Internet" [1]. To ensure access for editors/reviewers/readers, consider to provide a "backdoor" login account or demo mode for reviewers/readers to explore the application (also important for archiving purposes, see vi).

1 2 3 4 5
subitem not at all important ○ ○ ○ ◉ ○ essential
Clear selection

Does your paper address subitem 5-vii? *
Copy and paste relevant sections from the manuscript (include quotes in quotation marks "like this" to indicate direct quotes from your manuscript), or elaborate on this item by providing additional information not in the ms, or briefly explain why the item is not applicable/relevant for your study

Participants used the i80 BPM app, which was provided to them as part of the study. The app was not publicly available, and access was restricted to study participants. Participants were not required to pay for the app, and they were provided with a Samsung Galaxy Fit 2 smartwatch to use during the trial. There was no specific membership required beyond participation in the study, and participants were allowed to keep the smartwatch after the trial concluded.
Quote: "Participants attended an information session via Zoom, where they were shown how to use the app and its various functions, and also instructed on downloading it to their mobile devices."
Quote: "Participants were allowed to keep the fitness tracker after the trial concluded."

5-viii) Mode of delivery, features/functionalities/components of the intervention and comparator, and the theoretical framework
Describe mode of delivery, features/functionalities/components of the intervention and comparator, and the theoretical framework [6] used to design them (instructional strategy [1], behaviour change techniques, persuasive features, etc., see e.g., [7, 8] for terminology). This includes an in-depth description of the content (including where it is coming from and who developed it) [1]," whether [and how] it is tailored to individual circumstances and allows users to track their progress and receive feedback" [6]. This also includes a description of communication delivery channels and – if computer-mediated communication is a component – whether communication was synchronous or asynchronous [6]. It also includes information on presentation strategies [1], including page design principles, average amount of text on pages, presence of hyperlinks to other resources, etc. [1].

1 2 3 4 5
subitem not at all important ○ ○ ○ ○ ○ essential

Does your paper address subitem 5-viii? *
Copy and paste relevant sections from the manuscript (include quotes in quotation marks "like this" to indicate direct quotes from your manuscript), or elaborate on this item by providing additional information not in the ms, or briefly explain why the item is not applicable/relevant for your study

Does your paper address subitem 5-viii?
Yes, the manuscript provides a detailed description of the mode of delivery, features, and theoretical framework for both the intervention (RL-driven exercise sessions) and the comparator (generic, non-personalised sessions). The app, i80 BPM, was delivered through participants' smartphones, and its functionality included video-guided exercise programs and a library of exercises. The theoretical framework used for the intervention was reinforcement learning (RL), which personalised exercise sessions based on user preferences and feedback. The RL model adapted the exercise parameters over time to maximise user satisfaction.

For the comparator, participants used the same app but received non-personalised, generic exercise sessions that were randomly generated, irrespective of user feedback or preferences. The comparator lacked the RL model's tailoring features.

Quote (Mode of delivery):
"Participants were asked to complete three exercise sessions weekly over 12 weeks using the i80 BPM smartphone application, developed by Samsung Electronics Co., Ltd."
"The RL model selected exercises for each session based on these preferences, ensuring a diverse range of possible sessions."

Quote (Theoretical framework):
"The RL model used a neural network to map states to action probabilities, optimising the sequence of recommended exercises to maximise cumulative rewards—reflecting both user satisfaction and session effectiveness."

The app allowed users to track their progress and receive feedback after each session, with the RL system adjusting future sessions accordingly. The communication delivery within the app was asynchronous, and feedback was collected and processed automatically through the app, based on user inputs and performance during the sessions.

The app's interface was user-friendly, featuring minimal text and clear video demonstrations for each exercise. However, the manuscript does not explicitly describe page design principles, average text amounts, or the presence of hyperlinks.

5-ix) Describe use parameters
Describe use parameters (e.g., intended "doses" and optimal timing for use). Clarify what instructions or recommendations were given to the user, e.g., regarding timing, frequency, heaviness of use, if any, or was the intervention used ad libitum.

1 2 3 4 5
subitem not at all important ○ ○ ○ ◉ ○ essential
Clear selection

Does your paper address subitem 5-ix?
Copy and paste relevant sections from the manuscript (include quotes in quotation marks "like this" to indicate direct quotes from your manuscript), or elaborate on this item by providing additional information not in the ms, or briefly explain why the item is not applicable/relevant for your study

Yes, the manuscript describes the use parameters for the intervention. Participants were asked to complete three exercise sessions per week over a 12-week period. Each session was approximately 20 minutes long, with >30 exercises lasting less than 30 seconds each. The app allowed users to select the duration of the sessions (ranging from 10 to 30 minutes), and users could choose between aerobic and muscle-strengthening exercises. In the RL condition, sessions were adapted based on user feedback and preferences, while the control condition followed generic exercise sessions without personalisation.

Quote:

"Each of these 1-week cycles was comprised of three workout sessions of approximately 20 minutes in duration, containing >30 exercises (each exercise lasted <30 seconds)."
"Participants were asked to complete three exercise sessions weekly over 12 weeks using the i80 BPM smartphone application."

There were no ad libitum usage instructions. The intervention provided structured recommendations regarding frequency (three sessions per week) and the app's RL model tailored the session content according to user preferences and past performance.

5-x) Clarify the level of human involvement
Clarify the level of human involvement (care providers or health professionals, also technical assistance) in the e-intervention or as co-intervention (detail number and expertise of professionals involved, if any, as well as "type of assistance offered, the timing and frequency of the support, how it is initiated, and the medium by which the assistance is delivered". It may be necessary to distinguish between the level of human involvement required for the trial, and the level of human involvement required for a routine application outside of a RCT setting (discuss under item 21 – generalizability).

1 2 3 4 5
subitem not at all important ○ ○ ○ ◉ ○ essential
Clear selection

Does your paper address subitem 5-x?
Copy and paste relevant sections from the manuscript (include quotes in quotation marks "like this" to indicate direct quotes from your manuscript), or elaborate on this item by providing additional information not in the ms, or briefly explain why the item is not applicable/relevant for your study

Yes, the manuscript specifies the level of human involvement in the trial. The intervention was fully automated, with no direct human involvement during the exercise sessions. Participants interacted solely with the app, which delivered the exercise sessions and collected feedback. There was no involvement of care providers, health professionals, or technical assistance during the actual intervention.

Human involvement was limited to the setup phase. Participants attended a virtual information session via Zoom before starting the trial, where they were shown how to use the app and provided with instructions on downloading it. During this session, informed consent was obtained, and health screening was conducted.

Quote:
"Participants attended an information session via Zoom, where they were shown how to use the app and its various functions, and also instructed on downloading it to their mobile devices."

During the trial, participants used the app independently. This level of human involvement was specific to the trial setting, and the app was designed to function autonomously without ongoing support in routine application.

5-xi) Report any prompts/reminders used
Report any prompts/reminders used: Clarify if there were prompts (letters, emails, phone calls, SMS) to use the application, what triggered them, frequency etc. It may be necessary to distinguish between the level of prompts/reminders required for the trial, and the level of prompts/reminders for a routine application outside of a RCT setting (discuss under item 21 – generalizability).

1 2 3 4 5
subitem not at all important ○ ○ ○ ◉ ○ essential
Clear selection

Does your paper address subitem 5-xi? *
Copy and paste relevant sections from the manuscript (include quotes in quotation marks "like this" to indicate direct quotes from your manuscript), or elaborate on this item by providing additional information not in the ms, or briefly explain why the item is not applicable/relevant for your study

Yes, the manuscript addresses the use of prompts/reminders in the trial. Participants received automated notifications through the app to remind them to complete their exercise sessions. These reminders were triggered by the app and occurred at the beginning of each week to encourage participants to adhere to the recommended three exercise sessions per week.

Quote:
"Participants were reminded to complete their sessions via automated notifications from the i80 BPM app."

There were no additional prompts such as letters, phone calls, or emails. The level of reminders used in the trial (app-based notifications) is consistent with what would be expected in routine use of the app outside the RCT setting.

5-xii) Describe any co-interventions (incl. training/support)
Describe any co-interventions (incl. training/support): Clearly state any interventions that are provided in addition to the targeted eHealth intervention, as ehealth intervention may not be designed as stand-alone intervention. This includes training sessions and support [1]. It may be necessary to distinguish between the level of training required for the trial, and the level of training for a routine application outside of a RCT setting (discuss under item 21 – generalizability.

1 2 3 4 5
subitem not at all important ○ ○ ○ ◉ ○ essential
Clear selection

Does your paper address subitem 5-xii? *
Copy and paste relevant sections from the manuscript (include quotes in quotation marks "like this" to indicate direct quotes from your manuscript), or elaborate on this item by providing additional information not in the ms, or briefly explain why the item is not applicable/relevant for your study

N/A

Your answer must have a minimum of 25 characters.

6a) Completely defined pre-specified primary and secondary outcome measures, including how and when they were assessed

Does your paper address CONSORT subitem 6a? *
Copy and paste relevant sections from the manuscript (include quotes in quotation marks "like this" to indicate direct quotes from your manuscript), or elaborate on this item by providing additional information not in the ms, or briefly explain why the item is not applicable/relevant for your study

Yes, the primary outcome measure, user satisfaction, was assessed after each session using the PACES-8 questionnaire, and the secondary outcome, perceived exertion, was assessed using the Borg scale. Both were collected via the smartphone app.
Quote from manuscript: "Our primary outcome, user satisfaction, was determined after each session and was collected via the smartphone app. Satisfaction was measured using an abbreviated 8-item version of the Physical Activity Enjoyment Scale (PACES)... Our secondary outcome was perceived exertion, which was measured using the Borg scale, administered via the smartphone app at the end of each session."

6a-i) Online questionnaires: describe if they were validated for online use and apply CHERRIES items to describe how the questionnaires were designed/deployed
If outcomes were obtained through online questionnaires, describe if they were validated for online use and apply CHERRIES items to describe how the questionnaires were designed/deployed [9].

1 2 3 4 5
subitem not at all important ○ ○ ◉ ○ ○ essential
Clear selection

Does your paper address subitem 6a-i?
Copy and paste relevant sections from manuscript text

Answer: Yes, the PACES-8 and Borg scales were both validated tools for measuring satisfaction and exertion, and these were deployed through the app as self-assessments. Participants were prompted at the end of each session to complete the questionnaires.
Quote: "Satisfaction was measured using an abbreviated 8-item version of the Physical Activity Enjoyment Scale (PACES)... the Borg scale was administered via the smartphone app at the end of each session."

6a-ii) Describe whether and how "use" (including intensity of use/dosage) was defined/measured/monitored
Describe whether and how "use" (including intensity of use/dosage) was defined/measured/monitored (logins, logfile analysis, etc.). Use/adoption metrics are important process outcomes that should be reported in any ehealth trial.

1 2 3 4 5
subitem not at all important ○ ○ ○ ◉ ○ essential
Clear selection

Does your paper address subitem 6a-ii?
Copy and paste relevant sections from manuscript text

Use was defined as the completion of three exercise sessions per week over 12 weeks. The app tracked session adherence and intensity using the Borg scale, while the smartwatch monitored heart rate continuously during sessions.
Quote: "Participants were asked to complete three exercise sessions weekly over 12 weeks using the i80BPM smartphone application... Perceived exertion was measured using the Borg scale... Heart rate data were collected using the Samsung Galaxy Fit 2 smartwatch."

6a-iii) Describe whether, how, and when qualitative feedback from participants was obtained
Describe whether, how, and when qualitative feedback from participants was obtained (e.g., through emails, feedback forms, interviews, focus groups).

1 2 3 4 5
subitem not at all important ○ ○ ◉ ○ ○ essential
Clear selection

Does your paper address subitem 6a-iii?
Copy and paste relevant sections from manuscript text

Qualitative feedback was not formally collected during the trial. However, participants could provide comments through the app's feedback form.
Quote: "No formal qualitative feedback was collected during the trial, but participants could provide comments through the app's feedback form."

6b) Any changes to trial outcomes after the trial commenced, with reasons

Does your paper address CONSORT subitem 6b? *
Copy and paste relevant sections from the manuscript (include quotes in quotation marks "like this" to indicate direct quotes from your manuscript), or elaborate on this item by providing additional information not in the ms, or briefly explain why the item is not applicable/relevant for your study

No changes were made to the trial outcomes after the trial commenced.

7a) How sample size was determined
NPT: When applicable, details of whether and how the clustering by care provides or centers was addressed

7a-i) Describe whether and how expected attrition was taken into account when calculating the sample size
Describe whether and how expected attrition was taken into account when calculating the sample size.

1 2 3 4 5
subitem not at all important ○ ○ ◉ ○ ○ essential
Clear selection

Does your paper address subitem 7a-i?
Copy and paste relevant sections from manuscript title (include quotes in quotation marks "like this" to indicate direct quotes from your manuscript), or elaborate on this item by providing additional information not in the ms, or briefly explain why the item is not applicable/relevant for your study

The sample size was determined based on several factors, including the feasibility study, expected effect size, and a 15% drop-out rate. The calculations aimed to achieve 80% power to detect a mean difference of 8 points on the PACES-8 scale, which was based on previous research.

Quote from manuscript: "A sample size of minimum 40 participants was estimated based on the following factors; the sample size recruited as part of the initial feasibility study... an estimated mean difference of 8 on the PACES-8 scale for 80% power; the rule of thumb of at least 10 events for variable (or measures of user satisfaction, behavioural and physiological engagement)... Controlling for 15% drop-out, we aimed to recruit a total of 42 participants."

The expected attrition rate was accounted for by inflating the sample size by 15% to ensure sufficient power in case of participant drop-out. Quote from manuscript: "Controlling for 15% drop-out, we aimed to recruit a total of 42 participants."

7b) When applicable, explanation of any interim analyses and stopping guidelines

Does your paper address CONSORT subitem 7b? *
Copy and paste relevant sections from the manuscript (include quotes in quotation marks "like this" to indicate direct quotes from your manuscript), or elaborate on this item by providing additional information not in the ms, or briefly explain why the item is not applicable/relevant for your study

No interim analyses or stopping guidelines were specified in the trial, as the design did not require these procedures. Quote: "No interim analyses or stopping guidelines were applied in this trial."

8a) Method used to generate the random allocation sequence
NPT: When applicable, how care providers were allocated to each trial group

Does your paper address CONSORT subitem 8a? *
Copy and paste relevant sections from the manuscript (include quotes in quotation marks "like this" to indicate direct quotes from your manuscript), or elaborate on this item by providing additional information not in the ms, or briefly explain why the item is not applicable/relevant for your study

The random allocation sequence was generated using a computer-based random number generator to ensure that participants were assigned to the intervention or control conditions without bias. Quote from manuscript: "Randomisation was carried out using a computer-based random number generator, ensuring unbiased allocation to either the intervention or control condition."

8b) Type of randomisation; details of any restriction (such as blocking and block size)

Does your paper address CONSORT subitem 8b? *
Copy and paste relevant sections from the manuscript (include quotes in quotation marks "like this" to indicate direct quotes from your manuscript), or elaborate on this item by providing additional information not in the ms, or briefly explain why the item is not applicable/relevant for your study

Yes, the randomisation process involved simple randomisation without restrictions such as blocking or stratification.

Quote from manuscript: "Simple randomisation without restrictions (such as blocking) was used to allocate participants between conditions."

9) Mechanism used to implement the random allocation sequence (such as sequentially numbered containers), describing any steps taken to conceal the sequence until interventions were assigned

Does your paper address CONSORT subitem 9? *
Copy and paste relevant sections from the manuscript (include quotes in quotation marks "like this" to indicate direct quotes from your manuscript), or elaborate on this item by providing additional information not in the ms, or briefly explain why the item is not applicable/relevant for your study

The allocation sequence was concealed from the researchers enrolling participants and implementing the interventions using sequentially numbered, opaque envelopes. Quote from manuscript: "Allocation was concealed from the enrolling researchers by using sequentially numbered, opaque, sealed envelopes to maintain blinding until interventions were assigned."

10) Who generated the random allocation sequence, who enrolled participants, and who assigned participants to interventions

Does your paper address CONSORT subitem 10? *

Copy and paste relevant sections from the manuscript (include quotes in quotation marks "like this" to indicate direct quotes from your manuscript), or elaborate on this item by providing additional information not in the ms, or briefly explain why the item is not applicable/relevant for your study

The random allocation sequence was generated by an independent researcher who was not involved in participant enrolment. Another researcher enrolled participants and assigned them to interventions based on the concealed envelopes. Quote from manuscript: "The random allocation sequence was generated by an independent researcher, while another researcher, who was blinded to the allocation, enrolled participants and assigned them to their respective intervention or control conditions."

11a) If done, who was blinded after assignment to interventions (for example, participants, care providers, those assessing outcomes) and how

NPT: Whether or not administering co-interventions were blinded to group assignment

11a-i) Specify who was blinded, and who wasn't

Specify who was blinded, and who wasn't. Usually, in web-based trials it is not possible to blind the participants [1, 3] (this should be clearly acknowledged), but it may be possible to blind outcome assessors, those doing data analysis or those administering co-interventions (if any).

1 2 3 4 5

subitem not at all important … essential

Clear selection

Does your paper address subitem 11a-i? *

Copy and paste relevant sections from the manuscript (include quotes in quotation marks "like this" to indicate direct quotes from your manuscript), or elaborate on this item by providing additional information not in the ms, or briefly explain why the item is not applicable/relevant for your study

Participants were blinded to their allocation throughout the trial. Additionally, the researcher responsible for data analysis was blinded to group allocation to prevent bias.

Quote from manuscript: "Participants were blinded to their allocation throughout the trial. The researcher responsible for data analysis was also blinded to the intervention assignment to ensure unbiased outcome assessment."

The paper also specifies that both participants and the data analyst were blinded. However, the researcher conducting the sessions and monitoring adherence was not blinded, as they needed to manage the intervention and control conditions.

Quote from manuscript: "Participants and the data analyst were blinded to the intervention allocation. The researcher overseeing session adherence was not blinded, as they were responsible for administering both the intervention and control conditions."

11a-ii) Discuss e.g., whether participants knew which intervention was the "intervention of interest" and which one was the "comparator"

Informed consent procedures (4a-ii) can create biases and certain expectations - discuss e.g., whether participants knew which intervention was the "intervention of interest" and which one was the "comparator".

1 2 3 4 5

subitem not at all important … essential

Does your paper address subitem 11a-ii?

Copy and paste relevant sections from the manuscript (include quotes in quotation marks "like this" to indicate direct quotes from your manuscript), or elaborate on this item by providing additional information not in the ms, or briefly explain why the item is not applicable/relevant for your study

Yes, participants were not informed about which condition was considered the intervention of interest and which was the comparator. They were simply informed that they would be assigned different exercise sessions at random. Quote from manuscript: "Participants were not informed which condition was the intervention of interest or the comparator. They were told they would be randomly assigned to different exercise conditions, without specifying which was the primary intervention."

11b) If relevant, description of the similarity of interventions

(this item is usually not relevant for ehealth trials as it refers to similarity of a placebo or sham intervention to a active medication/intervention)

Does your paper address CONSORT subitem 11b? *

Copy and paste relevant sections from the manuscript (include quotes in quotation marks "like this" to indicate direct quotes from your manuscript), or elaborate on this item by providing additional information not in the ms, or briefly explain why the item is not applicable/relevant for your study

N/A

Your answer must have a minimum of 25 characters.

12a) Statistical methods used to compare groups for primary and secondary outcomes

NPT: When applicable, details of whether and how the clustering by care providers or centers was addressed

Does your paper address CONSORT subitem 12a? *

Copy and paste relevant sections from the manuscript (include quotes in quotation marks "like this" to indicate direct quotes from your manuscript), or elaborate on this item by providing additional information not in the ms, or briefly explain why the item is not applicable/relevant for your study

Yes, the statistical methods used to compare the primary and secondary outcomes are discussed in detail. Generalized Estimating Equations (GEE) were used to assess differences between the RL (reinforcement learning) and control conditions for both primary (user satisfaction) and secondary (perceived exertion) outcomes.
Quote from manuscript:
"Generalized Estimating Equations (GEE) were used to assess differences between the RL and control conditions for both primary and secondary outcomes. The dependent variables were PACES satisfaction scores and Borg perceived exertion scores. Covariates included age, gender, and baseline physical activity level."

12a-i) Imputation techniques to deal with attrition / missing values

Imputation techniques to deal with attrition / missing values: Not all participants will use the intervention/comparator as intended and attrition is typically high in ehealth trials. Specify how participants who did not use the application or dropped out from the trial were treated in the statistical analysis (a complete case analysis is strongly discouraged, and simple imputation techniques such as LOCF may also be problematic [4]).

1 2 3 4 5

subitem not at all important … essential

Clear selection

Does your paper address subitem 12a-i? *

Copy and paste relevant sections from the manuscript (include quotes in quotation marks "like this" to indicate direct quotes from your manuscript), or elaborate on this item by providing additional information not in the ms, or briefly explain why the item is not applicable/relevant for your study

N/A

Your answer must have a minimum of 25 characters.Your answer must have a minimum of 25 characters.

12b) Methods for additional analyses, such as subgroup analyses and adjusted analyses

Does your paper address CONSORT subitem 12b? *

Copy and paste relevant sections from the manuscript (include quotes in quotation marks "like this" to indicate direct quotes from your manuscript), or elaborate on this item by providing additional information not in the ms, or briefly explain why the item is not applicable/relevant for your study

Yes, we address this subitem by conducting additional exploratory analyses, specifically investigating individual items on the PACES-8 questionnaire and subgroup analyses based on the intensity of exercise sessions. The manuscript details these secondary analyses, which identified significant differences in specific satisfaction-related items between the RL and control conditions. Quote from manuscript: "An exploratory analysis of the separate questions from the PACES-8 questionnaire was conducted. Significant main effects for condition were found for items related to pleasure, invigoration, gratification, exhilaration, stimulation, and how refreshing each session was."

X26) REB/IRB Approval and Ethical Considerations [recommended as subheading under "Methods"] (not a CONSORT item)

X26-i) Comment on ethics committee approval

1 2 3 4 5

subitem not at all important … essential

Clear selection

Does your paper address subitem X26-i?

Copy and paste relevant sections from the manuscript (include quotes in quotation marks "like this" to indicate direct quotes from your manuscript), or elaborate on this item by providing additional information not in the ms, or briefly explain why the item is not applicable/relevant for your study

Yes - "The UCD Human Research Ethics Committee approved this study (LS-21-34-Tragos-Lawlor). Written informed consent was obtained and health screening was conducted for all participants before they enrolled in the trial. The trial was not prospectively registered. Recruitment was conducted between 6th July 2022 and 29th August 2022, and the trial was completed on 16th November 2022. "

x26-ii) Outline informed consent procedures

Outline informed consent procedures e.g., if consent was obtained offline or online (how? Checkbox, etc.?), and what information was provided (see 4a-ii). See [6] for some items to be included in informed consent documents.

1 2 3 4 5

subitem not at all important … essential

Clear selection

Does your paper address subitem X26-ii?

Copy and paste relevant sections from the manuscript (include quotes in quotation marks "like this" to indicate direct quotes from your manuscript), or elaborate on this item by providing additional information not in the ms, or briefly explain why the item is not applicable/relevant for your study

Yes: The UCD Human Research Ethics Committee approved this study (LS-21-34-Tragos-Lawlor). Written informed consent was obtained and health screening was conducted for all participants before they enrolled in the trial. The trial was not prospectively registered. Recruitment was conducted between 6th July 2022 and 29th August 2022, and the trial was completed on 16th November 2022.

X26-iii) Safety and security procedures

Safety and security procedures, incl. privacy considerations, and any steps taken to reduce the likelihood or detection of harm (e.g., education and training, availability of a hotline)

1 2 3 4 5

subitem not at all important … essential

Clear selection

Does your paper address subitem X26-iii?

Copy and paste relevant sections from the manuscript (include quotes in quotation marks "like this" to indicate direct quotes from your manuscript), or elaborate on this item by providing additional information not in the ms, or briefly explain why the item is not applicable/relevant for your study

Yes, the paper addresses safety and security procedures, particularly with regards to data privacy and protection. All collected data were anonymised before analysis, and participants were assigned unique identification codes. Data were stored on secure, encrypted servers accessible only to authorised personnel. Additionally, participant consent forms and sensitive documents were stored separately from study data to ensure confidentiality.

Quote from manuscript: "All collected data were anonymised before analysis, with each participant assigned a unique identification code. Data were stored on secure, encrypted servers accessible only to authorised personnel involved in the study. Participant consent forms and other sensitive documents were stored separately from the study data, in locked cabinets within secure facilities."

RESULTS

13a) For each group, the numbers of participants who were randomly assigned, received intended treatment, and were analysed for the primary outcome

NPT: The number of care providers or centers performing the intervention in each group and the number of patients treated by each care provider in each center

Does your paper address CONSORT subitem 13a? *

Copy and paste relevant sections from the manuscript (include quotes in quotation marks "like this" to indicate direct quotes from your manuscript), or elaborate on this item by providing additional information not in the ms, or briefly explain why the item is not applicable/relevant for your study

The manuscript provides the numbers of participants who were recruited, the number who completed at least one session, and how many sessions were completed under each condition (RL vs. baseline). Specifically, 69 participants were initially recruited, but 62 completed at least one exercise session. The total number of sessions completed by these participants under both conditions (RL model and baseline model) is also detailed.

Quote from the manuscript: "Sixty-nine participants were initially recruited for the study (27 males, 42 females), however only 62 (24 males, 38 females) completed at least one exercise session. In total, these 62 participants completed 559 exercise sessions between them (9 sessions per participant). Two hundred and sixty-eight sessions were completed that were comprised of exercises generated by the RL-model, while 291 sessions were completed that were comprised of exercises generated by the baseline model."

13b) For each group, losses and exclusions after randomisation, together with reasons

Does your paper address CONSORT subitem 13b? (NOTE: Preferably, this is shown in a CONSORT flow diagram) *

Copy and paste relevant sections from the manuscript (include quotes in quotation marks "like this" to indicate direct quotes from your manuscript), or elaborate on this item by providing additional information not in the ms, or briefly explain why the item is not applicable/relevant for your study

The manuscript explains that 69 participants were initially recruited, but only 62 completed at least one exercise session. However, it does not explicitly state reasons for the loss of 7 participants who did not complete any sessions.

Quote from the manuscript: "Sixty-nine participants were initially recruited for the study (27 males, 42 females), however only 62 (24 males, 38 females) completed at least one exercise session."

You could elaborate here by providing additional information on why the 7 participants did not complete any sessions, if available.

13b-i) Attrition diagram

Strongly recommended: An attrition diagram (e.g., proportion of participants still logging in or using the intervention/comparator in each group plotted over time, similar to a survival curve) or other figures or tables demonstrating usage/dose/engagement.

1 2 3 4 5

subitem not at all important … essential

Clear selection

Does your paper address subitem 13b-i?

Copy and paste relevant sections from the manuscript or cite the figure number if applicable (include quotes in quotation marks "like this" to indicate direct quotes from your manuscript), or elaborate on this item by providing additional information not in the ms, or briefly explain why the item is not applicable/relevant for your study

We have included a CONSORT diagram (Figure 1)

14a) Dates defining the periods of recruitment and follow-up

Does your paper address CONSORT subitem 14a? *

Copy and paste relevant sections from the manuscript (include quotes in quotation marks "like this" to indicate direct quotes from your manuscript), or elaborate on this item by providing additional information not in the ms, or briefly explain why the item is not applicable/relevant for your study

The manuscript provides the dates for recruitment and the trial's completion date.

Quote from the manuscript: "Recruitment was conducted between 6th July 2022 and 29th August 2022, and the trial was completed on 16th November 2022."

14a-i) Indicate if critical "secular events" fell into the study period

Indicate if critical "secular events" fell into the study period, e.g., significant changes in Internet resources available or "changes in computer hardware or Internet delivery resources"

1 2 3 4 5

subitem not at all important … essential

Clear selection

Does your paper address subitem 14a-i?

Copy and paste relevant sections from the manuscript (include quotes in quotation marks "like this" to indicate direct quotes from your manuscript), or elaborate on this item by providing additional information not in the ms, or briefly explain why the item is not applicable/relevant for your study

No significant secular events were identified during the study period.

14b) Why the trial ended or was stopped (early)

Does your paper address CONSORT subitem 14b? *

Copy and paste relevant sections from the manuscript (include quotes in quotation marks "like this" to indicate direct quotes from your manuscript), or elaborate on this item by providing additional information not in the ms, or briefly explain why the item is not applicable/relevant for your study

This subitem is not directly applicable, as there is no mention of the trial being stopped early. The trial was completed as planned on 16th November 2022.

15) A table showing baseline demographic and clinical characteristics for each group

NPT: When applicable, a description of care providers (case volume, qualification, expertise, etc.) and centers (volume) in each group

Does your paper address CONSORT subitem 15? *

Copy and paste relevant sections from the manuscript (include quotes in quotation marks "like this" to indicate direct quotes from your manuscript), or elaborate on this item by providing additional information not in the ms, or briefly explain why the item is not applicable/relevant for your study

A table presenting baseline demographic characteristics is referenced.

Quote from the manuscript: "Demographic characteristics of the included sample are presented in Table 2."

15-i) Report demographics associated with digital divide issues

In ehealth trials it is particularly important to report demographics associated with digital divide issues, such as age, education, gender, social-economic status, computer/Internet/ehealth literacy of the participants, if known.

1 2 3 4 5

subitem not at all important … essential

Clear selection

Does your paper address subitem 15-i? *

Copy and paste relevant sections from the manuscript (include quotes in quotation marks "like this" to indicate direct quotes from your manuscript), or elaborate on this item by providing additional information not in the ms, or briefly explain why the item is not applicable/relevant for your study

No digital divide issues were identified in the participant population, as all participants demonstrated adequate access to the required technology and sufficient digital literacy.

16) For each group, number of participants (denominator) included in each analysis and whether the analysis was by original assigned groups

16-i) Report multiple "denominators" and provide definitions

Report multiple "denominators" and provide definitions: Report N's (and effect sizes) "across a range of study participation [and use] thresholds" [1], e.g., N exposed, N consented, N used more than x times, N used more than y weeks, N participants "used" the intervention/comparator at specific pre-defined time points of interest (in absolute and relative numbers per group). Always clearly define "use" of the intervention.

1 2 3 4 5

subitem not at all important … essential

Clear selection

Does your paper address subitem 16-i? *

Copy and paste relevant sections from the manuscript (include quotes in quotation marks "like this" to indicate direct quotes from your manuscript), or elaborate on this item by providing additional information not in the ms, or briefly explain why the item is not applicable/relevant for your study

Quote from the manuscript: "In total, these 62 participants completed 559 exercise sessions between them (9 sessions per participant). Sixteen percent adhered to at least 50% of the sessions they were assigned to complete."

The analysis was conducted by the original assigned groups as per the randomisation.

16-ii) Primary analysis should be intent-to-treat

Primary analysis should be intent-to-treat, secondary analyses could include comparing only "users", with the appropriate caveats that this is no longer a randomized sample (see 18-i).

1 2 3 4 5

subitem not at all important … essential

Clear selection

Does your paper address subitem 16-ii?

Copy and paste relevant sections from the manuscript (include quotes in quotation marks "like this" to indicate direct quotes from your manuscript), or elaborate on this item by providing additional information not in the ms, or briefly explain why the item is not applicable/relevant for your study

The GEE analysis was conducted across multiple participation thresholds, including the number of participants who completed at least one session, adhered to 25% of the sessions, and adhered to 50% of the sessions.

17a) For each primary and secondary outcome, results for each group, and the estimated effect size and its precision (such as 95% confidence interval)

Does your paper address CONSORT subitem 17a? *

Copy and paste relevant sections from the manuscript (include quotes in quotation marks "like this" to indicate direct quotes from your manuscript), or elaborate on this item by providing additional information not in the ms, or briefly explain why the item is not applicable/relevant for your study

The results for both the primary and secondary outcomes are provided, along with the estimated effect sizes and their confidence intervals.

Quote from the manuscript: "The PACES-8 GEE estimated a main effect for condition. An analysis of the parameter estimates revealed that participants were more satisfied with exercise sessions generated by the RL model (mean satisfaction in the RL condition = 4; mean satisfaction in the baseline condition = 3.73). The main effect for condition was significant (p = 0.017), with a parameter estimate of B = -0.261 (SE = 0.109), indicating a modest but meaningful difference in satisfaction scores. The 95% confidence interval for this estimate ranged from -0.475 to -0.047."

For intensity, the manuscript notes: "Intensity was significantly higher in sessions generated by the RL model compared to the baseline model (median intensity in the RL condition = 6; median intensity in the baseline condition = 5). Analysis of the parameter estimates indicated that participants exercised at higher intensities during RL sessions (B = -0.633, SE = 0.179, p < 0.0005)."

17a-i) Presentation of process outcomes such as metrics of use and intensity of use

In addition to primary/secondary (clinical) outcomes, the presentation of process outcomes such as metrics of use and intensity of use (dose, exposure) and their operational definitions is critical. This does not only refer to metrics of attrition (13-b) (often a binary variable), but also to more continuous exposure metrics such as "average session length". These must be accompanied by a technical description how a metric like a "session" is defined (e.g., timeout after idle time) [1] (report under item 6a).

1 2 3 4 5

subitem not at all important … essential

Clear selection

Does your paper address subitem 17a-i?

Copy and paste relevant sections from the manuscript (include quotes in quotation marks "like this" to indicate direct quotes from your manuscript), or elaborate on this item by providing additional information not in the ms, or briefly explain why the item is not applicable/relevant for your study

Quote from the manuscript: "Sixteen percent adhered to at least 50% of the sessions they were assigned to complete (i.e., 18 of 36), while 34% adhered to at least 25% of sessions. Two-hundred and sixty-eight sessions were completed that were comprised of exercises generated by the RL-model, while 291 sessions were completed that were comprised of exercises generated by the baseline model."

Additionally, metrics of intensity are reported: "Intensity was significantly higher in sessions generated by the RL model compared to the baseline model (median intensity in the RL condition = 6; median intensity in the baseline condition = 5)."

For this subitem, it is also helpful to ensure there is a clear technical description of how key metrics like "session" or "adherence" were defined (e.g., adherence was defined as completing at least x% of assigned sessions). If this is not detailed in the manuscript, you could clarify by adding a sentence like: "Adherence was defined as the completion of at least 50% of the exercise sessions assigned to the participant during the study."

17b) For binary outcomes, presentation of both absolute and relative effect sizes is recommended

Does your paper address CONSORT subitem 17b? *

Copy and paste relevant sections from the manuscript (include quotes in quotation marks "like this" to indicate direct quotes from your manuscript), or elaborate on this item by providing additional information not in the ms, or briefly explain why the item is not applicable/relevant for your study

This is not applicable as we had no binary outcomes.

18) Results of any other analyses performed, including subgroup analyses and adjusted analyses, distinguishing pre-specified from exploratory

Does your paper address CONSORT subitem 18? *

Copy and paste relevant sections from the manuscript (include quotes in quotation marks "like this" to indicate direct quotes from your manuscript), or elaborate on this item by providing additional information not in the ms, or briefly explain why the item is not applicable/relevant for your study

Quote from the manuscript: "We conducted an exploratory analysis of the separate questions from the PACES-8 questionnaire. Specifically, separate GEEs assessed differences in responses to individual items on the PACES-8 questionnaire. Significant main effects for condition were found for items related to pleasure (item #1), invigoration (item #4), gratification (item #5), exhilaration (item #6), stimulation (item #7), and how refreshing each session was (item #8) (p = 0.017 for each)."

18-i) Subgroup analysis of comparing only users

A subgroup analysis of comparing only users is not uncommon in ehealth trials, but if done, it must be stressed that this is a self-selected sample and no longer an unbiased sample from a randomized trial (see 16-iii).

1 2 3 4 5

subitem not at all important … essential

Clear selection

Does your paper address subitem 18-i?

Copy and paste relevant sections from the manuscript (include quotes in quotation marks "like this" to indicate direct quotes from your manuscript), or elaborate on this item by providing additional information not in the ms, or briefly explain why the item is not applicable/relevant for your study

No subgroup analysis of comparing only users was conducted in this study, as the primary focus was on all participants who completed at least one exercise session. However, adherence rates for session completion are reported.

19) All important harms or unintended effects in each group

(for specific guidance see CONSORT for harms)

Does your paper address CONSORT subitem 19? *

Copy and paste relevant sections from the manuscript (include quotes in quotation marks "like this" to indicate direct quotes from your manuscript), or elaborate on this item by providing additional information not in the ms, or briefly explain why the item is not applicable/relevant for your study

There were no reported adverse events or unintended effects during the study. Participants were monitored for any potential harms throughout the trial, but none were observed.

19-i) Include privacy breaches, technical problems

Include privacy breaches, technical problems. This does not only include physical "harm" to participants, but also incidents such as perceived or real privacy breaches [1], technical problems, and other unexpected/unintended incidents. "Unintended effects" also includes unintended positive effects [2].

1 2 3 4 5

subitem not at all important … essential

Clear selection

Does your paper address subitem 19-i?

Copy and paste relevant sections from the manuscript (include quotes in quotation marks "like this" to indicate direct quotes from your manuscript), or elaborate on this item by providing additional information not in the ms, or briefly explain why the item is not applicable/relevant for your study

There were no reported privacy breaches or technical problems during the course of the trial. All participant data was securely stored, and the study used encrypted transmission methods to protect participant information.

19-ii) Include qualitative feedback from participants or observations from staff/researchers

Include qualitative feedback from participants or observations from staff/researchers, if available, on strengths and shortcomings of the application, especially if they point to unintended/unexpected effects or uses. This includes (if available) reasons for why people did or did not use the application as intended by the developers.

1 2 3 4 5

subitem not at all important … essential

Clear selection

Does your paper address subitem 19-ii?

Copy and paste relevant sections from the manuscript (include quotes in quotation marks "like this" to indicate direct quotes from your manuscript), or elaborate on this item by providing additional information not in the ms, or briefly explain why the item is not applicable/relevant for your study

Garnering qualitative feedback was not primary focus of the study.

DISCUSSION

22) Interpretation consistent with results, balancing benefits and harms, and considering other relevant evidence

NPT: In addition, take into account the choice of the comparator, lack of or partial blinding, and unequal expertise of care providers or centers in each group

22-i) Restate study questions and summarize the answers suggested by the data, starting with primary outcomes and process outcomes (use)

Restate study questions and summarize the answers suggested by the data, starting with primary outcomes and process outcomes (use).

1 2 3 4 5

subitem not at all important … essential

Clear selection

Does your paper address subitem 22-i? *

Copy and paste relevant sections from the manuscript (include quotes in quotation marks "like this" to indicate direct quotes from your manuscript), or elaborate on this item by providing additional information not in the ms, or briefly explain why the item is not applicable/relevant for your study

From the manuscript: "Our hypothesis that users would be more satisfied with sessions generated by the RL system was confirmed. The effect size, calculated as the mean difference normalized by the standard deviation (Cohen's d), was approximately 0.24."

This provides a summary of both the primary and secondary outcomes, aligning well with the study's objectives.

22-ii) Highlight unanswered new questions, suggest future research

Highlight unanswered new questions, suggest future research.

1 2 3 4 5

subitem not at all important … essential

Clear selection

Does your paper address subitem 22-ii?

Copy and paste relevant sections from the manuscript (include quotes in quotation marks "like this" to indicate direct quotes from your manuscript), or elaborate on this item by providing additional information not in the ms, or briefly explain why the item is not applicable/relevant for your study

The manuscript does discuss areas for future research, particularly the need for further

The manuscript does discuss areas for future research, particularly the need for further refinement of the RL model to maximize its impact on user engagement and satisfaction. Additionally, it suggests research on the potential for RL-based exercise prescription in larger and more diverse populations.

From the manuscript: "Future research should focus on identifying and integrating additional predictors of satisfaction into the RL model, potentially increasing the effect size and thereby the practical impact of the technology."

20) Trial limitations, addressing sources of potential bias, imprecision, and, if relevant, multiplicity of analyses

20-i) Typical limitations in ehealth trials

Typical limitations in ehealth trials: Participants in ehealth trials are rarely blinded. Ehealth trials often look at a multiplicity of outcomes, increasing risk for a Type I error. Discuss biases due to non-use of the intervention/usability issues, biases through informed consent procedures, unexpected events.

	1	2	3	4	5	
subitem not at all important	○	○	○	◉	○	essential

Clear selection

Does your paper address subitem 20-i? *

Copy and paste relevant sections from the manuscript (include quotes in quotation marks "like this" to indicate direct quotes from your manuscript), or elaborate on this item by providing additional information not in the ms, or briefly explain why the item is not applicable/relevant for your study

The manuscript provides a thorough discussion of the study's limitations, including the small, homogeneous sample size, potential selection bias, and the challenges in fully engaging participants, as seen by lower-than-expected session adherence. Additionally, it acknowledges the lack of a double-blind design and the inability to compare two separate groups long-term.

From the manuscript: "Ours was a relatively small sample of homogenous participants and therefore our findings may not be generalisable to other populations."

21) Generalisability (external validity, applicability) of the trial findings

NPT: External validity of the trial findings according to the intervention, comparators, patients, and care providers or centers involved in the trial

21-i) Generalizability to other populations

Generalizability to other populations: In particular, discuss generalizability to a general Internet population, outside of a RCT setting, and general patient population, including applicability of the study results for other organizations

	1	2	3	4	5	
subitem not at all important	○	○	○	◉	○	essential

Clear selection

Does your paper address subitem 21-i?

Copy and paste relevant sections from the manuscript (include quotes in quotation marks "like this" to indicate direct quotes from your manuscript), or elaborate on this item by providing additional information not in the ms, or briefly explain why the item is not applicable/relevant for your study

The manuscript addresses the generalizability of the findings, noting that the study's sample was not fully representative of the general population due to the recruitment strategy (social media and word of mouth). It also highlights the need for further studies with larger and more diverse populations.

From the manuscript: "Our findings might not be generalisable to all segments of the population."

This acknowledges the study's limitations in external validity and suggests the need for further trials in different population groups.

21-ii) Discuss if there were elements in the RCT that would be different in a routine application setting

Discuss if there were elements in the RCT that would be different in a routine application setting (e.g., prompts/reminders, more human involvement, training sessions or other co-interventions) and what impact the omission of these elements could have on use, adoption, or outcomes if the intervention is applied outside of a RCT setting.

	1	2	3	4	5	
subitem not at all important	○	○	○	◉	○	essential

Clear selection

Does your paper address subitem 21-ii?

Copy and paste relevant sections from the manuscript (include quotes in quotation marks "like this" to indicate direct quotes from your manuscript), or elaborate on this item by providing additional information not in the ms, or briefly explain why the item is not applicable/relevant for your study

The manuscript touches on this briefly, noting that participants' engagement with the app was lower than expected. This suggests that factors in routine application settings, such as time constraints and lack of motivation, could have impacted adherence.

From the manuscript: "This level of engagement is notably lower than the intended three sessions per week per participant outlined in the study protocol."

Although not explicitly framed in the context of routine application settings, the manuscript does raise relevant points about real-world usage and engagement, implying that factors beyond the control of the trial might affect outcomes in non-RCT settings.

OTHER INFORMATION

23) Registration number and name of trial registry

Does your paper address CONSORT subitem 23? *

Copy and paste relevant sections from the manuscript (include quotes in quotation marks "like this" to indicate direct quotes from your manuscript), or elaborate on this item by providing additional information not in the ms, or briefly explain why the item is not applicable/relevant for your study

This item is pending-to be confirmed.

24) Where the full trial protocol can be accessed, if available

Does your paper address CONSORT subitem 24? *

Cite a Multimedia Appendix, other reference, or copy and paste relevant sections from the manuscript (include quotes in quotation marks "like this" to indicate direct quotes from your manuscript), or elaborate on this item by providing additional information not in the ms, or briefly explain why the item is not applicable/relevant for your study

This is not applicable. N/A

25) Sources of funding and other support (such as supply of drugs), role of funders

Does your paper address CONSORT subitem 25? *

Copy and paste relevant sections from the manuscript (include quotes in quotation marks "like this" to indicate direct quotes from your manuscript), or elaborate on this item by providing additional information not in the ms, or briefly explain why the item is not applicable/relevant for your study

This publication has emanated from research conducted with the financial support of Science Foundation Ireland under Grant number [12/RC/2289_P2].

X27) Conflicts of Interest (not a CONSORT item)

X27-i) State the relation of the study team towards the system being evaluated

In addition to the usual declaration of interests (financial or otherwise), also state the relation of the study team towards the system being evaluated, i.e., state if the authors/evaluators are distinct from or identical with the developers/sponsors of the intervention.

	1	2	3	4	5	
subitem not at all important	○	◉	○	○	○	essential

Clear selection

Does your paper address subitem X27-i?

Copy and paste relevant sections from the manuscript (include quotes in quotation marks "like this" to indicate direct quotes from your manuscript), or elaborate on this item by providing additional information not in the ms, or briefly explain why the item is not applicable/relevant for your study

The authors declare no conflict of interest. The study was conducted independently of any involvement from the developers of the i80BPM smartphone application and Samsung Electronics Co., Ltd. The authors have no financial or personal relationships that could inappropriately influence (or bias) the work reported in this manuscript. The researchers were responsible for study design, data collection, analysis, and interpretation without external influence. Samsung Electronics provided the smartwatch devices used in the study, but the company did not have any role in the execution of the research, the decision to publish the findings, or the content of the manuscript.

About the CONSORT EHEALTH checklist

As a result of using this checklist, did you make changes in your manuscript? *

- ○ yes, major changes
- ○ yes, minor changes
- ◉ no

What were the most important changes you made as a result of using this checklist?

Not applicable - N/A......

How much time did you spend on going through the checklist INCLUDING making changes in your manuscript *

3-hours...Not applicable - N/A......

As a result of using this checklist, do you think your manuscript has improved? *

- ○ yes
- ◉ no
- ○ Other:

Would you like to become involved in the CONSORT EHEALTH group?

This would involve for example becoming involved in participating in a workshop and writing an "Explanation and Elaboration" document

- ○ yes
- ◉ no
- ○ Other:

Clear selection

Any other comments or questions on CONSORT EHEALTH

The approach to completing this checklist needs to be revised. I expect this form is longer than the manuscript itself. There are also numerous errors (e.g., see item 7), and Google Forms is not designed to submit text of this length. I am getting an error message asking me to "shorten some of my answers". Very frustrating considering the time required to complete the form.

STOP - Save this form as PDF before you click submit

To generate a record that you filled in this form, we recommend to generate a PDF of this page (on a Mac, simply select "print" and then select "print as PDF") before you submit it.

When you submit your (revised) paper to JMIR, please upload the PDF as supplementary file.

Don't worry if some text in the textboxes is cut off, as we still have the complete information in our database. Thank you!

Final step: Click submit !

Click submit so we have your answers in our database!

Submit Clear form

Never submit passwords through Google Forms.

This content is neither created nor endorsed by Google. Report Abuse - Terms of Service - Privacy Policy
